# Design and Synthesis of a Library of Lead-Like 2,4-Bisheterocyclic Substituted Thiophenes as Selective Dyrk/Clk Inhibitors

**DOI:** 10.1371/journal.pone.0087851

**Published:** 2014-03-27

**Authors:** Christian Schmitt, Dagmar Kail, Marica Mariano, Martin Empting, Nadja Weber, Tamara Paul, Rolf W. Hartmann, Matthias Engel

**Affiliations:** 1 Department of Pharmaceutical and Medicinal Chemistry, Saarland University, Saarbrücken, Germany; 2 PharmBioTec GmbH, Saarbrücken, Germany; 3 Department of Drug Design and Optimization, Helmholtz-Institut für Pharmazeutische Forschung Saarland, Saarbrücken, Germany; University of Pittsburgh School of Medicine, United States of America

## Abstract

The Dyrk family of protein kinases is implicated in the pathogenesis of several diseases, including cancer and neurodegeneration. Pharmacological inhibitors were mainly described for Dyrk1A so far, but in fewer cases for Dyrk1B, Dyrk2 or other isoforms. Herein, we report the development and optimization of 2,4-bisheterocyclic substituted thiophenes as a novel class of Dyrk inhibitors. The optimized hit compounds displayed favorable pharmacokinetic properties and high ligand efficiencies, and inhibited Dyrk1B in intact cells. In a larger selectivity screen, only Clk1 and Clk4 were identified as additional targets of compound **48**, but no other kinases frequently reported as off-targets. Interestingly, Dyrk1A is implicated in the regulation of alternative splicing, a function shared with Clk1/Clk4; thus, some of the dual inhibitors might be useful as efficient splicing modulators. A further compound (**29**) inhibited Dyrk1A and 1B with an IC_50_ of 130 nM, showing a moderate selectivity over Dyrk2. Since penetration of the central nervous system (CNS) seems possible based on the physicochemical properties, this compound might serve as a lead for the development of potential therapeutic agents against glioblastoma. Furthermore, an inhibitor selective for Dyrk2 (**24**) was also identified, which might be are suitable as a pharmacological tool to dissect Dyrk2 isoform–mediated functions.

## Introduction

The Dyrk family of kinases belongs to the CMGC superfamily and comprises five members, Dyrk1A, 1B, 2, 3, 4A and 4B [1]. The name is an abbreviation for “dual-specificity tyrosine-(Y)-phosphorylation regulated kinase”, based on the observation that autophosphorylation at a tyrosine residue in the activation loop is required for the activation of the kinase, while all observed substrate phosphorylations proceed at serine/threonine residues [2].

Dyrk1A was identified as a major kinase phosphorylating the microtubule–associated tau protein, often functioning as a priming kinase for glycogen-synthase kinase (GSK)3β [3]–[6]. Hyperphosphorylation of tau protein is believed to be one of the triggering factors for neurodegeneration because it leads to the formation of neurotoxic neurofibrillary tangles [7], [8]. In particular, Dyrk1A is discussed to be causally involved in the development of Alzheimer–like neurodegenerative diseases in Down Syndrome patients, where the kinase is 1.5-fold overexpressed due to its location in the so-called Down Syndrome Critical Region on chromosome 21 [5], [9], [10]. An additional pathogenic mechanism contributing to the development of tauopathies in Down Syndrome is the altered splicing of tau protein pre-mRNA which results in an imbalance between 3R-tau and 4R-tau isoforms. This imbalance is caused by the increased phosphorylation of the alternative splicing factor (ASF) and of the Serine/Arginine-rich Protein 55 (SRp55) by Dyrk1A that leads to a reduced inclusion of tau exon 10 [11]–[15].

Skipping of tau exon 10 was also reported to be enhanced through the action of cdc-like kinase 1 (Clk1) [16], a dual specificity kinase from the CMGC kinase group, which is often affected by Dyrk1A inhibitors and *vice versa* [17]–[21]. Interestingly, both kinases appear to phosphorylate SRp55, suggesting that both might be involved in the pathogenesis of tauopathies [16].

The most closely related isoform, Dyrk1B, is rather ubiquitously expressed, but particularly strong in skeletal muscle tissue and in several types of cancer [22], [23]. One of the known physiological roles of Dyrk1B is the regulation of muscle cell regeneration after damage [24], [25]. In tumors, Dyrk1B exerts an anti-apoptotic function as it mediates some of the survival signals activated by the K-Ras oncoprotein [26]. In addition, Dyrk1B inhibits cell cycle progression in G0/G1 in some tumor cells by phosphorylation of p27Kip1. As a consequence, this protein is retained in the nucleus, where it inhibits cyclin–dependent kinase (CDK)2 [27], [28]. Depletion of cyclin D1 by targeting the protein to proteosomal degradation is another mechanism by which Dyrk1B blocks the cell cycle [29], a function which might be shared with Dyrk1A [30], [31]. Hence, tumor cells are maintained in quiescence, allowing them to escape the eradication by chemotherapeutic agents [32], [33]. In addition, both Dyrk1A and 1B (but not Dyrk2) are functionally linked with E3 ubiquitin ligases [34]–[36], suggesting that they may target the same proteins for proteosomal degradation as part of a fail-safe mechanism. In virus–induced cancers, Dyrk1A may play a role as an important anti-apoptotic factor, as has been demonstrated in HPV16–immortalized keratinocytes and cervical lesions [37]. Malignant cervical lesions contain significantly more Dyrk1A than normal tissue. Importantly, a recent study validated Dyrk1A as a new target in EGFR–dependent glioblastoma: inhibition of Dyrk1A promoted degradation of EGFR and sharply decreased tumor cell growth and viability [38]. Altogether, there is increasing evidence that inhibition of Dyrk1B, possibly in combination with Dyrk1A, might represent a promising, yet underexploited anti-tumor strategy.

The reported roles of Dyrk2 in the tumor biology appear rather controversial. The corresponding gene was originally identified as the most frequently amplified and overexpressed gene in lung adenocarcinomas and esophageal carcinoma [39]. In addition, overexpression of Dyrk2 was found to be associated with tumor progression in gastrointestinal stromal tumors [40]. However, more recent studies point to a reduced or abolished Dyrk2 expression in multiple human tumor types, which correlates with invasiveness in the case of human breast cancer [41]. In non-small cell lung carcinomas, Dyrk2 expression was identified as a marker that correlated with induction of apoptosis in response to chemotherapeutic treatment [42], which is possibly mediated through co-activation of the p53 tumor suppressor protein [43]. Furthermore, abrogation of Dyrk2 expression had been shown to result in the activation of telomerase activity [44], and in the stabilization of the c-Jun and c-Myc proto-oncogenes [41]. However, in the light of all these potential tumor suppressor activities it is fair to ask why Dyrk2 is strongly overexpressed in several tumor entities. This question could effectively be addressed using selective small molecule inhibitors.

In general, relatively few reports were published on the remaining Dyrk isoforms. Dyrk3 has also been shown to attenuate apoptosis in response to cytokine withdrawal in hematopoietic cells of erythroid lineage [45], [46]. Future studies will show if it might be a potential pharmacological target in lymphomas, since a profiling of tyrosine phosphorylation in cancer cells identified Dyrk3 (and Dyrk1A) as prominently tyrosine –phosphorylated proteins in an anaplastic large cell lymphoma cell line, suggesting that these kinases might be abnormally activated in lymphoma cells [47].

Dyrk4A and 4B are the least studied of all Dyrk isoforms; no substrate has been discovered so far for these kinases. Dyrk4 was recently identified in a neuronal overexpression screen as a kinase which increased the number of dendritic branches in hippocampal neurons [48].

Among the published Dyrk inhibitors, the natural product harmine proved to be most useful due to its selectivity for Dyrk1A and – with 3-fold lower potency – for Dyrk1B [49]. However, it is an even more potent inhibitor of monoamine oxidase A, impeding its applicability for studies in the brain [38], [50], [51]. Furthermore, some controversy exists in literature on potential genotoxic effects of harmine, that were attributed to its flat tricyclic structure which might intercalate DNA [52]–[54]. Several further inhibitor classes were published which potently inhibited Dyrk1A. However, these compounds were either not selective for the Dyrk family, or no selectivity data were provided [49], [55]–[60]. While it can be challenging to develop selective ATP-competitive kinase inhibitors, the example of harmine has demonstrated that in the case of the Dyrk isoforms, this might be achievable using very small, compact molecules. In contrast to the situation with Dyrk1A, only few small molecule inhibitors were reported for the other Dyrk family members. A pyrido[2,3-d]pyrimidine derivative was published as a Dyrk1B inhibitor; however it was three times more potent toward Dyrk1A and also inhibited microtubule affinity–regulating kinase (MARK)1 in a small counter screen [32]. This series was extended in a recent report, however, selectivity data or evidence for an inhibition of the target kinases in cells were not provided [61]. Some acridine analogs were found to potently inhibit Dyrk2, with only few kinases being affected outside the CMGC family [62].

In the present report, we describe the design and synthesis of a focused library of a new class of dual Dyrk/Clk1/4 inhibitors, which displayed a high degree of selectivity over other kinase families. The new compounds exhibited high ligand efficiencies and favorable physicochemical properties. Moreover, evidence for an inhibition of Dyrk1B in intact cells was provided by several cell-based assays.

## Materials and Methods

### Biology

Dyrk1B and Clk1 were purchased from Life Technologies (LOT # 877059G, Catalog # PV4649 and LOT # 943590A, Catalog # PV3315). Casein kinase 2 (CK2) substrate peptide was purchased from Millipore (LOT # JBC1949760, Catalog # 12-330); cOmplete Mini Protease inhibitor cocktail tablets were purchased from Roche. Woodtide substrate peptide and RS repeat substrate peptide were custom synthesized at the Department of Medical Biochemistry and Molecular Biology, Saarland University, Homburg, Germany.

### Protein expression and purification

An expression plasmid encoding the catalytic domain of human Dyrk1A fused to an N-terminal hexahistidine-tag (termed pET45b-Dyrk1A-cd) was constructed as described in the Supporting Information section (Construction of the pET45b-Dyrk1A-cd expression plasmid, in File S2). Human hexahistidine-tagged Dyrk1A (His_6_-Dyrk1A) and glutathione S-tranferase (GST)-fusioned Dyrk2 were both expressed in *Escherichia coli*. *E. coli* BL21(DE3) cells were co-transformed using either the pET45b-Dyrk1A-cd or the pGEX-2TK-Dyrk2 (kind gift from W. Becker, Aachen) expression plasmid together with the pRARE plasmid (Novagen), carrying genes of human tRNAs which are rare in *E. coli* to increase the yield of recombinant proteins. The transformed bacteria were grown in LB medium containing 50 µg/mL ampicillin and 25 µg/mL chloramphenicol. Protein expression was induced by addition of 0.5 mM isopropyl β-D-thiogalactopyranoside (IPTG) overnight at 18°C. Cell pellets were resuspended in lysis buffer (50 mM Tris/HCl, pH 7.4, 0.27 M Sucrose, 1 mM sodium orthovanadate, 10 mM β-glycerophosphate disodium salt, 1 mM DTT, 50 mM NaF, 1% Triton X100, cOmplete Mini Protease inhibitor cocktail tablets) and lysed by sonication. His_6_-Dyrk1A was purified by affinity chromatography using Ni^2+^-Sepharose beads (GE Healthcare Bio Sciences, LOT # 10038389) as follows: the cleared cell lysate was gently stirred with the beads overnight at 4°C. Then the beads were filled into an empty chromatography column and the column washed three times with 10 volumes lysis buffer, followed by one wash with lysis buffer containing 20 mM imidazole. After another wash using 50 mM Tris/HCl, pH 7.2, and 100 mM NaCl, the bound proteins were eluted using 50 mM Tris/HCl, pH 7.2, 100 mM NaCl, 1 mM DTT, 200 mM imidazole, and 0.1 mM EGTA. The proteins were dialyzed against the same buffer without imidazole, 20% glycerol was added, and the proteins snap frozen in dry ice/isopropanol and stored at −80°C. GST-Dyrk2 fusion protein was purified from the lysate using glutathione-agarose beads (Machery-Nagel, LOT # 1212001) essentially as described previously for GST-PKCζ [63].

### Kinase assays

Dyrk1A, Dyrk1B, Dyrk2, CK2α and Clk1 kinase reactions were performed in a reaction buffer containing 50 mM Tris/HCl, pH 7.4, 0.1 mM EGTA, 0.5 mM DTT, 10 mM MgCl_2_, 100 µM ATP and 0.33 µM [γ-^32^ATP] as well as the appropriate substrate peptides, which were 100 µM Woodtide for the Dyrk isoforms (KKISGRLSPIMTEQ-NH_2_), 60 µM CK2 substrate peptide (RRRDDDSDDD-NH_2_, from Millipore) for CK2α or RS repeat peptide (GRSRSRSRSRSRSRSR) for Clk1. The CK2α protein was a kind gift of M. Montenarh (Homburg). The kinase reactions were performed at 30°C for 15 min and terminated by spotting 5 µL of the reaction mixture onto a P81 phosphocellulose membrane (Whatman). The membrane was washed four times with 0.3% phosphoric acid and one time with acetone and dried. The dry membrane was exposed in a cassette to a Phosphor Screen Imaging Plate (FujiFilm) and the signals detected by scanning of the imaging plate in a Fuji FLA-3000 PhosphoImager. The spots were quantified using AIDA software (Raytest, Version 3.52) to determine the activities of the kinases in the assay reactions. For IC_50_ determinations, eight concentrations of each compound were used in triplicates, and the percentage of inhibition at 5 µM was also calculated from the average of triplicate values. IC_50_ values were calculated by fitting the data with Origin Pro 8.6 (OriginLabs). The calculated IC_50_ values are representative of at least two independent determinations.

### ROS Assay

U2OS osteosarcoma cells were plated on six well cell culture plates (5*10^5^ cells/well). The cells were starved for 24 hours in McCoys medium containing 0.5% fetal calf serum (FCS) and incubated for 48 hours with four different concentrations of compound **29** in McCoys medium also containing 0.5% FCS. The final DMSO concentration in each well was 0.1%. Cells were washed with phosphate–buffered saline (PBS) and incubated for 30 min at 37°C in the dark with 10 µM dihydroethidium (DHE) in PBS and washed with PBS. The cells were then trypsinized, collected by centrifugation, and lysed with lysis buffer (100 mM Tris/HCl, pH 7.3, 2 mM EGTA, 2% Triton X100). The amount of ethidium produced by reactive oxygen species (ROS) was quantified by measuring the fluorescence (Ex485, Em620) in a POLARstar plate reader (BMG Labtech, Offenburg, Germany). Values obtained for different compound concentrations were compared using the two-sided Student's t-test; levels of significance thus obtained are indicated by asterisks.

### Determination of logP, pk_a_ and logS parameters

The physicochemical parameters were determined on a Sirius T3 machine (Sirius Analytical machines, East Sussex, UK) by automated titration according to the manufacturer's instructions.

### Caspase-3 Assay

U2OS osteosarcoma cells were seeded in a 96 well flat bottom plate (15*10^3^ cells/well). The cells were starved for at least 24 hours in McCoys medium containing 0.5% FCS and incubated for 48 hours with four different concentrations of **11**, **29** and **48** in McCoys medium containing 0.5% FCS. The induction of caspase-3/7 activity was measured using the Promega Caspase-Glo 3/7 assay system (Promega, LOT 0000054568). The resulting luciferase reaction signals that are proportional to the caspase induction were measured in a POLARstar plate reader (BMG Labtech, Offenburg, Germany). In a control experiment using purified luciferase and substrate, we verified that the tested compounds did not inhibit the luciferase enzymatic activity itself. Values obtained for different compound concentrations were compared using the two-sided Student's t test; levels of significance thus obtained are indicated by asterisks.

### Real-Time PCR

U2OS cells were seeded in 6 well flat bottom plates (5*10^5^ cells/well) and grown to confluency. The cells were then starved for 48 hours in McCoys medium containing 0.5% FCS and incubated for 72 hours with compounds **29** and **48** or DMSO in McCoys medium containing 0.5% FCS; the medium containing the compounds or DMSO, respectively, was refreshed each day, and each concentration applied in triplicates. The cells were then harvested and total RNA isolated using the RNeasy Mini Kit (Qiagen, Cat. No. 74104). 1 µg of total RNA was transcribed to cDNA using the QuantiTect Rev. Transcription Kit (Qiagen, Cat. No. 205311), and 20 ng of cDNA used per Real-Time (RT)-PCR experiment (assuming quantitative reverse transcription). RT-PCR was performed in a StepOnePlus Real-Time PCR System (Life Technologies) using the SYBR green RT-PCR Kit (Peqlab, Cat. No. 07-KK4603-01) according to the manufacturer's protocol, using the following cycling conditions: initial denaturation: 95°C, 40 sec, followed by 45 cycles of denaturation at 95°C, 2 sec, and annealing/extension at 60°C, 40 sec. The following primer pairs were used: β-actin: 5′-TGC GTG ACA TTA AGG AGA AG-3′ and 5′-GTC AGG CAG CTC GTA GCT CT-3′ [64]; CDH4: 5′-CAA CCT GAA CGC CAT CAA CAT C-3′ and 5′-CGC AAG CTG AGT TGG GCA TAG-3′ [65]; FGF2: 5′-CAA GCG GCT GTA CTG CAA AAA-3′ and 5′-GTT CGT TTC AGT GCC ACA TAC-3′ [66]; BIM: 5′-TCA GCG CCT TTG TGA GGA G-3′ and 5′-CAG GCA AGG ATC AGG TAG GTG-3′ (PrimerBank ID: 50593007c2, [67]); TRADD: 5′-GCT GTT TGA GTT GCA TCC TAG C-3′ and 5′-CCG CAC TTC AGA TTT CGC A-3′ (PrimerBank ID: 115387096c1, [67]); FasL: 5′-TGC CTT GGT AGG ATT GGG C-3′ and 5′-GCT GGT AGA CTC TCG GAG TTC-3′ (PrimerBank ID: 4557328c1, [67]); SOD2: 5′-CGA CCT GCC CTA CGA CTA CG-3′ and 5′-TGA CCA CCA CCA TTG AAC TT-3′ [68]; CP: 5′-CCC TGG AGA ATG GAT GCT CA-3′ and 5′-CTA ACA TGC TTC CCA CGG ATA TT-3′ [69].

### ViaLight Toxicity Assay

V79 hamster lung fibroblast cells were plated at a density of 45,000 cells per well in a white 96 well plate and allowed to adhere and grow for 24 hours. The compounds were then applied as dilutions in cell culture medium (DMEM with 10% FCS and penicillin/streptomycin mix) at a final concentration of 5, 10, and 20 µM, respectively. The plate was further incubated for 48 hours at 37°C in a humidified atmosphere containing 5% CO_2_. The ATP level was detected with a LONZA ViaLight™ Plus Kit according to protocol 1 of the vendor's instructions. Luminescence was measured in a POLARstar plate reader (BMG Labtech, Offenburg, Germany). The background luminescence was obtained from equally treated, cell-free wells containing serum, and subtracted from all values. Inhibition values obtained for different compound concentrations were compared using the two-sided Student's t-test; levels of significance thus obtained are indicated by asterisks.

### Metabolic Stability Assay

The assay was performed with liver microsomes from male Sprague-Dawley rats (BD Bioscience, Catalog # 452501) and half-lives of the compounds calculated essentially as previously described [70], except that the compounds were used at a final concentration of 0.5 µM. The samples were analyzed by LC-MS/MS analysis on a TSQ Quantum Access MAX (Thermo Fisher Scientific) using 0.5 µM amitriptyline as an internal standard. The microsomal intrinsic clearance (Cl_int_) estimates were calculated according to Obach [71] using the formula Cl_int_ = 0,69314718/t_1/2 microsomal_) * (mL incubation volume/mg microsomal protein) * (mg microsomal protein/g liver) * (g liver/kg body weight). In the assay, the incubation volume was 0.2 ml, containing 0.045 mg microsomal protein, and the following literature values for male Sprague-Dawley rats were used for the calculation: 23.3 mg microsomal protein/g liver, 8.0 g liver weight, 180 g body weight [72]. From the Cl_int_ values, the blood clearance rate was calculated using the formula Cl_blood_ = Q * (1−e∧(−Cl_int_/Q)) [71] with Q = 55.2 ml/min/kg (rat hepatic blood flow, taken from Ref. [73]). This formula disregards serum and microsomal protein binding events, however it had given the best predictions for weakly basic and neutral compounds [71].

### Docking studies

The multi-step *in silico* experiment employing local docking and molecular dynamics simulations was performed with YASARA structure using a self–written command sequence (macro) with the AMBER03 force field [74]–[76]. First, the crystal structure of Dyrk1A in complex with harmine (PDB accession code: 3ANR) was loaded into the software. Then, the ligand was removed and a grid box of approximately 9 nm^3^ was set up around the active site of the enzyme for the subsequent local docking experiment using the built-in AutoDock 4 algorithm [77]. Compound **29** possesses two rotatable bonds which link both heterocyclic substituents to the thiophene core. This rather small degree of rotational freedom was systematically sampled via 100 conformers using 10 rotamers for each bond. Each of these structures was subjected to a rigid docking experiment to the prepared Dyrk1A enzyme structure using 999 individual docking runs.

The ten best ligand-enzyme structures of the previous step were then simulated in 0.9% (m/v) NaCl (aq.) at 298 K and pH 7.4 for 50 µsec with fixed backbone atoms. After energy minimization, the resulting complexes were subjected to a final rigid local docking experiment using a smaller grid box which extended 1 Å around the ligand atoms. Ligand–receptor interactions of this structure as well as the parent Dyrk1A–harmine complex were analyzed with MOE 2010 and images were rendered with POVRay.

### Chemistry

#### General chemical methods

Chemical starting material was purchased from Sigma-Aldrich, CombiBlocks or Alfa Aesar and used without further purification. Synthesis of the focused library was performed by an ISYNTH robotic platform from Chemspeed Technologies. Purity of the compounds was determined using an Agilent 1100 series HPLC system from Agilent Technologies, a GC Trace Ultra from Thermo or a Waters autopurification system from Waters Corporation. The purity of the compounds used in the biological assays was ≥95%. Mass spectra (ESI) were measured on an AB Sciex Qtrap2000 from AB Sciex or a Waters 3100 Mass detector from Waters Corporation. Mass spectra (EI) were measured on a DSQ II from Thermo. ^1^H and ^13^C NMR spectra were recorded on either a Bruker DRX-500 (^1^H, 500 MHz; ^13^C, 126 MHz) instrument at 300 K or on a Bruker Fourier300 (^1^H, 300 MHz; ^13^C, 75 MHz) NMR spectrometer at 300 K in the deuterated solvents indicated. IR spectra were recorded on a Perkin Elmer Spectrum 100 FT-IR spectrometer, Perkin Elmer, Rodgau, Germany. Flash column chromatography was performed using silica gel 60 (Merck, 35–70 µm). Reaction/flash monitoring was done by thin layer chromatography (TLC) on ALUGRAM SIL G/UV_254_ (Macherey-Nagel) employing UV detection.

*Procedure for the synthesis of 3-(4-bromothiophen-2-yl)pyridine* **i**: 1 g (8.14 mmol) of 3-Pyridylboronic acid was dissolved under nitrogen in 10 mL of dioxane and 4 mL of water. To this solution 1.97 g (8.14 mmol) of 2, 4-dibromothiophene, 0.376 g (0.325 mmol) of tetrakis(triphenylphosphine)Palladium(0) and 1.72 g (16.28 mmol) of sodium carbonate were added successively. The mixture was heated to reflux and the reaction progress was monitored by TLC. After completion of the reaction the crude product was washed with water and brine, dried over magnesium sulfate and purified by flash column chromatography eluting with ethyl acetate/hexane 1∶5 to yield 1.3 g (67%) of 3-(4-bromothiophen-2-yl)pyridine as a white solid. ^1^H NMR (500 MHz, Methanol-*d*_4_) δ (ppm) 7.41–7.51 (m, 3 H) 8.02 (dt, *J* = 7.96, 0.75 Hz, 1 H) 8.46–8.50 (m, 1 H) 8.76–8.81 (m, 1 H); ^13^C NMR (126 MHz, Methanol-*d*_4_) δ (ppm) 112.06, 125.02, 125.63, 128.51, 131.22, 134.89, 142.31, 146.92, 149.56; Purity (FID): 99%, t_R_: 6.69 min; MS (EI), *m/z* [M]^+^: 239.87 calc.: 238.94.

*Procedure for the synthesis of [5-(pyridin-3-yl)thiophen-3-yl]boronic acid* **ii**: To a solution of 2.64 g (11 mmol) of **i** in anhydrous toluene/THF under nitrogen atmosphere 3.3 mL (14.3 mmol) of triisopropyl borate were added followed by a careful addition of 5.72 mL (14.3 mmol) *n*-BuLi (2.5 M in hexanes) at −78°C (yellow to orange) over 45 min. The reaction was stirred at bath temperature for half an hour before adjusting the bath to −25°C. After 5 min 28.6 mmol of 2 M HCl were added and the mixture was stirred for half an hour at RT, before being transferred to a separatory funnel with 15 mL of water and 9 mL of THF. The organic layer was separated, extracted with 9 mL of water and the aqueous phases were adjusted to pH 7 with 5 M NaOH (2.1 mL, precipitation), before being extracted with THF (3×15 mL). The combined organic layers were dried (MgSO_4_) and concentrated. The crude crystals were directly used without further purification.

Procedure for the synthesis of *5-(4-bromothiophen-2-yl)-1,3-oxazole* **iii**: (Modified from Besselievre et al. 2008 [78]. To a solution of 2.5 g (13.1 mmol) of 4-bromo-2-thiophenecarbaldehyde in 10 mL of methanol, 2.81 g (14.4 mmol) tosylmethylisocyanate and 3.62 g (26.2 mmol) of potassium carbonate were added. The mixture was heated for 4 h to reflux. The solvent was evaporated in vacuo and the residue was poured into ice water. The precipitate was filtered off and dried. The crude product was recrystallized from hexane and directly used without further purification. ^1^H NMR (300 MHz, DMSO-*d*_6_) δ (ppm) 7.46–7.63 (m, 2H) 7.76 (s, 1H) 8.43 (s, 1 H), ^13^C NMR (75 MHz, DMSO-*d*_6_) δ (ppm) 110.33, 122.84, 124.61, 127.22, 130.98, 145.34, 152.16; Purity (FID): >99%, MS(EI), t_R_: 5.42 min, m/z [M]^+^: 228.77, calc. 228.92.

*Procedure for the synthesis of 5-(4-bromothiophen-2-yl)pyrimidine* **iv**: 0.62 g (5 mmol) of 5-pyrimidineboronic acid was diluted in a mixture of degassed dioxane/water (5∶1) under nitrogen atmosphere. To this solution 1.34 g (5 mmol) of 2,4-dibromothiophene, 0.289 g (0.25 mmol) of tetrakis(triphenylphosphine)palladium(0) and 1.59 g (15 mmol) of Na_2_CO_3_ were added successively. The mixture was heated to 90°C and stirred for two days. After completeness of the reaction the crude mixture was poured into water and extracted with diethyl ether. The combined organic layers were washed with brine, dried over Na_2_SO_4_, filtered off and the solvent was removed under reduced pressure. The crude product was purified by flash column chromatography eluting with ethyl acetate/cyclohexane (1∶3) to give 0.55 g (46%) of 5-(4-bromothiophen-2-yl)pyrimidine as yellow crystals. ^1^H-NMR (300 MHz, CDCl_3_): *δ* = 7.30–7.34 (m, 1H), 7.34–7.37 (m, 1H), 8.92 (d, *J* = 0.6 Hz, 2H), 9.17 (s, 1H). ^13^C–NMR (75 MHz, CDCl_3_): *δ* = 111.51, 124.33, 127.59, 127.85, 137.44, 153.44 (2C), 157.93; MS (ESI+): *m/z* (%) = 284 (13) [M+ACN+H^+^], 282 (12) [M+ACN+H^+^], 244 (13), 243 (100) [M+H^+^], 242 (12), 241 (100) [M+H^+^], calc. 240.94.

*Procedure for the synthesis of 2-(trimethylsilyl)-1,3-thiazole* **v**: 22.4 mL (56 mmol) of n-buthyllithium was added to 100 mL of dry diethyl ether and cooled to −78°C. Subsequently a solution of 8.2 g (50 mmol) of 2-bromothiazole in 80 mL of diethyl ether were added dropwise over 30 min and stirred for 30 min at −78°C. Then, a solution of 5.43 g of trimethylsilylchloride in 80 mL of diethyl ether was added dropwise and stirred for an additional hour at −78°C. The reaction mixture was allowed to warm to room temperature and saturated NaHCO_3_ solution (70 mL) was added. The organic phase was separated, dried over Na_2_SO_4_ and the solvent was removed under reduced pressure. The crude product was purified by vacuum distillation (0–10 mbar, bp 65–67°C) to yield 4.6 g (59%) of a colorless oil. ^1^H-NMR (300 MHz, CDCl_3_): *δ* = 0.42 (s, 9H), 7.53 (dd, *J* = 3.0, 0.7 Hz, 1H), 8.12 (d, *J* = 3.0 Hz, 1H), ^13^C-NMR (75 MHz, CDCl_3_): *δ* = −0.98, 121.34, 145.73, 174.37. Data are in accordance to Literature [79].

*Procedure for the synthesis of 5-(tetramethyl-1,3,2-dioxaborolan-2-yl)-1,3-thiazole* **vi**: A solution of 4.4 g (28 mmol) 2-(trimethylsilyl)-1,3-thiazole in dry THF was cooled to −78°C. Then, 13.4 mL (33.6 mmol) n-buthyllithium were added dropwise over 20 min. After 15 min, 6.32 g (33.6 mmol) of triisopropylborate were added dropwise and the solution was stirred for 90 min at −78°C. The solution was allowed to warm to room temperature and stirred for additional 30 min. Then 3.3 g (28 mmol) of pinacol, solved in 10 mL of dry THF was added and after 10 minutes the pH was adjusted to 5 with glacial acetic acid. The crude product was extracted with cyclohexane and dried under reduced pressure to yield 5.04 g of crude 5-(tetramethyl-1,3,2-dioxaborolan-2-yl)-1,3-thiazole. ^1^H-NMR (300 MHz, CDCl_3_): *δ* = 1.27–1.38 (m, 12H), 8.33 (s, 1H), 8.99 (s, 1H), ^13^C-NMR (75 MHz, CDCl_3_): *δ* = 24.69 (4C), 84.50 (2C), 152.57, 158.20. 1 C not det. NMR data are in accordance to literature [80].

*Procedure for the synthesis of 5-(4-bromothiophen-2-yl)-1,3-thiazole* **vii**: 0.95 g (4.5 mmol) of 5-(tetramethyl-1,3,2-dioxaborolan-2-yl)-1,3-thiazole were dissolved in a degassed mixture of dioxane/water (3∶1) under nitrogen atmosphere. To this solution 1.09 g (4.5 mmol) of 2,4 dibromothiophene, 0.29 g (0.25 mmol) of tetrakis(triphenylphosphine)palladium(0) and 1.22 g (11.5 mmol) of Na_2_CO_3_ were added and the reaction mixture was stirred for 2 days at 90°C. After completion of the reaction, the mixture was poured into water and extracted with diethyl ether. The combined organic layers were washed with water and brine, dried over Na_2_SO_4_ and the solvent was removed in vacuo. The crude product was purified by column flash chromatography eluting with ethyl acetate/cyclohexane (1∶10). ^1^H-NMR (300 MHz, CDCl_3_): *δ* = 7.12 (d, *J* = 1.4 Hz, 1H, Ar_m_-H), 7.19 (d, *J* = 1.4 Hz, 1H), 7.97 (s, 1H), 8.73 (d, *J* = 0.5 Hz, 1H). ^13^C-NMR (75 MHz, MeOD): *δ* = 110.57, 122.76, 128.22, 130.14, 133.48, 139.91, 152.27. Purity: ∼65%, *t*_R_ = 7.56 min; MS (ESI+): *m/z* (%) = 289 (17) [M+ACN+H^+^], 287 (14) [M+ACN+H^+^], 250 (20), 249 (23), 248 (100) [M+H^+^], 247 (2), 246 (100) [M+H^+^], calc. 245.90. The product was used for the synthesis of **48** without further purification.

*Procedure for the synthesis of compound* **4**: 152.4 mg (1.24 mmol) 3-pyridylboronic acid were dissolved under nitrogen in 10 mL dioxane and 2 mL water and stirred at room temperature. To this solution 150 mg (0.62 mmol) 2, 4-dibromothiophene, 72 mg (0.062 mmol) tetrakis(triphenylphosphine)palladium(0) and 265 mg (2.5 mmol) sodium carbonate were added successively. The mixture was stirred and heated to 100°C for 18 h. The reaction progress was monitored by TLC. After completion of the reaction, the mixture was cooled down to room temperature and the crude product was poured into water and extracted with diethyl ether (4×). The combined organic extracts were washed with water and brine, dried over magnesium sulfate and the solvent was removed in vacuo. The crude product was purified by flash column chromatography eluting with ethyl acetate and 1% Methanol to give 93 mg (63%) of **4** as a colorless solid. mp 110–111°C; IR (neat) 3047, 1469, 1125, 1022, 796, 696, 617 cm^−1^; ^1^H NMR (300 MHz, DMSO-*d*_6_) δ (ppm) 9.01 (dd, *J* = 0.75, 1.68 Hz, 1H), 8.94 (dd, *J* = 0.65, 2.33 Hz, 1H), 8.47–8.50 (m, 1H), 8.45–8.47 (m, 1H), 8.18 (d, *J* = 1.49 Hz, 1H), 8.10–8.16 (m, 1H), 8.04–8.10 (m, 2H), 7.42–7.45 (m, 1H), 7.38–7.42 (m, 1H); ^13^C NMR (75 MHz, DMSO-*d*_6_) δ (ppm) 123.30, 124.26, 124.37, 124.56, 129.94, 130.90, 133.14, 133.70, 139.78, 141.26, 146.69, 147.66, 148.84, 149.25; Purity (UV @254 nm): 99.1%, t_R_: min; MS (ESI), m/z [M+H]^+^: ; calc. 238,06

*Procedure for the synthesis of compound* **20**: 87 mg (0.42 mmol) of 3-bromoquinoline were dissolved in 10 mL of dry THF under nitrogen and the resulting solution was cooled to −78°C. To this solution 1.1 eq. (0.46 mmol, 184 µL) of n-buthyllithium was added in drops. The mixture was stirred for 1 hour at −78°C, then 1.2 eq. (0.5 mmol, 136 µL) of tributyl borate were added slowly and the mixture was stirred for 1.5 hours at −78°C. The mixture was allowed to warm up to room temperature. Then 2.5 eq. of Na_2_CO_3_ (1.3 mmol, 137 mg), 4 mol % of tetrakis(triphenylphosphine)palladium(0) (0.017 mmol, 19.4 mg), 100 mg (0.42 mmol) of 3-(4-bromothiophen-2-yl)pyridine and 4 mL of water were added successively and the mixture was heated to reflux overnight. The reaction progress was monitored by TLC analysis. The reaction was stopped and poured into water. The crude product was extracted with ethyl acetate and the combined organic layer was washed with water and brine, dried over magnesium sulfate and the solvent was removed in vacuo. The crude product was purified by flash column chromatography eluting with ethyl acetate/hexane 1∶5 to give 70 mg (58%) of **20** as a light yellow solid. mp 198–199°C; IR 3058, 1571, 1319, 1125, 1022, 798, 700, 615 cm^−1^; ^1^H NMR (500 MHz, Methanol–*d*_4_) δ (ppm) 7.36 (ddd, *J* = 7.90, 5.00, 0.95 Hz, 1H) 7.47–7.51 (m, 1 H) 7.62 (ddd, *J* = 8.35, 6.94, 1.42 Hz, 1 H) 7.84 (d, *J* = 7.88 Hz, 1 H) 7.86 (d, *J* = 1.58 Hz, 1 H) 7.89 (d, *J* = 8.51 Hz, 1 H) 7.92 (d, *J* = 1.26 Hz, 1 H) 8.01 (dt, *J* = 8.04, 1.81 Hz, 1 H) 8.36 (dd, *J* = 4.89, 1.42 Hz, 1 H) 8.45 (d, *J* = 1.89 Hz, 1 H) 8.78 (d, *J* = 2.21 Hz, 1 H) 9.07 (d, *J* = 2.21 Hz, 1 H); ^13^C NMR (126 MHz, Methanol–*d*_4_) δ (ppm) 124.00, 124.73, 125.61, 128.56, 128.95, 129.46, 129.69, 129.83, 130.93, 132.05, 133.99, 134.92, 140.87, 142.47, 146.98, 147.71, 149.15, 149.80; Purity (FID): 96.7%, t_R_: 10.15 min; MS (EI), *m/z* [M]^+^: 288.01 calc.: 288.072.

*General Procedure for the synthesis of focused libraries on the ISYNTH Chemspeed system*: The corresponding arylboronic acids/arylbromides (0.30 mmol) for diversification were manually prefilled into 20 mL disposable vials and placed on the ISYNTH. Cs_2_CO_3_ was added by an SDU (solid dosing unit). The interior of the vials was brought under protective gas atmosphere by repeated cycles of evacuation and flushing with argon. Water (0.75 mL) and stock solutions of the thiophene core (0.33 mmol in 1.25 mL of DMF) as well as Pd(dppf)Cl_2_ (0.015 mmol in 1.0 mL of DMF) were dispensed into each vial under a slight argon stream employing a 4-needle head attached to 10 mL syringes. Afterwards, the vials were automatically sealed and heated to 80°C under reflux for 15 h. After completion of the reaction 6 mL of water and 5 mL of diethyl ether were added to each vial. Solid matter was manually removed by filtration. The phase separation area was determined visually and the value (height in mm) entered. Separation of the organic phase and extraction of the aqueous one (twice with 6 mL of ethyl acetate) was performed by the ISYNTH robotic platform. MgSO_4_ was automatically added to the combined organic layers for drying and filtered off manually. The solvent was evaporated and the crude product was purified by preparative HPLC on a Waters Autopurification System employing a C-18 column (Waters X-Bridge OBD 19×150 mm, 5 µm) with a flow rate of 20 mL/min and respective 10 min gradients [solvents A (water+0.1% formic acid)/solvent B (acetonitrile/0.1% formic acid].

*3-methyl-5-[5-(pyridin-3-yl)thiophen-3-yl]pyridine* **5**: yield: 61%, mp 106–107°C; IR (neat) 3100, 1420, 1125, 1024, 807, 703, 613 cm^−1^; ^1^H-NMR (300 MHz, CDCl_3_): *δ* (ppm) 2.40 (s, 3H), 7.36 (dd, *J* = 7.9, 4.9 Hz, 1H) 7.52 (d, *J* = 1.3 Hz, 1H), 7.61 (d, *J* = 1.5 Hz, 1H), 7.72–7.77 (m, 1H), 7.92 (dt, *J* = 8.0, 2.0 Hz, 1H), 8.35–8.41 (m, 1H), 8.54 (dd, *J* = 4.8, 1.5 Hz, 1H), 8.69 (d, *J* = 2.1 Hz, 1H), 8.91 (d, *J* = 2.4 Hz, 1H). ^13^C-NMR (75 MHz, CDCl_3_): δ (ppm) 18.37, 122.10, 122.99, 123.89, 130.16, 131.02, 133.42, 133.74, 134.83, 139.55, 141.53, 143.60, 146.19, 147.84, 148.17; Purity(UV): 99%, t_R_: 3.28 MS (ESI+): *m/z* (%) = 255 (6), 254 (20), 253 (100) [M+H^+^], calc. 253.07.

*3-methoxy-5-[5-(pyridin-3-yl)thiophen-3-yl]pyridine* **8**: yield: 50%, mp 132–133°C; IR (neat) 3072, 1456, 1296, 1121, 1022, 799, 696, 612 cm^−1^; ^1^H NMR (300 MHz, Methanol-d_4_): *δ* (ppm) 8.85–8.93 (m, 1H), 8.42–8.54 (m, 2H), 8.07–8.20 (m, 2H), 7.93–7.99 (m, 1H), 7.85–7.92 (m, 1H), 7.65–7.74 (m, 1H), 7.47 (dd, *J* = 4.94, 6.99 Hz, 1H), 3.87–3.99 (m, 3H); ^13^C NMR (75 MHz, Methanol-d_4_): *δ* (ppm) 56.43, 119.75, 124.27, 124.87, 125.66, 132.13, 133.81, 135.00, 136.77, 139.96, 140.74, 142.38, 147.02, 149.16, 158.05. Purity (UV): 98%, *t*_R_ = 4.05 min; MS (ESI+): *m/z* (%) = 271 (21), 270 (57), 269 (100) [M+H^+^], calc. 269.07.

*3-fluoro-5-[5-(pyridin-3-yl)thiophen-3-yl]pyridine* **15**: yield: 21%, mp 186–187°C; IR (neat) 3069, 1417, 1185, 1142, 1023, 805, 697, 615 cm^−1^; ^1^H-NMR (300 MHz, CDCl_3_): *δ* (ppm) 8.92 (d, *J* = 2.42 Hz, 1H), 8.72 (t, *J* = 1.58 Hz, 1H), 8.57 (dd, *J* = 1.49, 4.84 Hz, 1H), 8.43 (d, *J* = 2.79 Hz, 1H), 7.87–7.95 (m, 1H), 7.57–7.63 (m, 2H), 7.55–7.57 (m, 1H), 7.35 (ddd, *J* = 0.75, 4.84, 8.01 Hz, 1H); ^13^C-NMR (75 MHz, CDCl_3_): δ (ppm) 120.11 (d, *J* = 18.7 Hz, 1C), 122.72, 122.75, 123.72, 129.74, 132.63 (d, *J* = 3.9 Hz, 1C), 133.03, 136.78 (d, *J* = 23.2 Hz, 1C), 138.51 (d, *J* = 3.9 Hz, 1C), 142.31, 143.36 (d, *J* = 4.0 Hz, 1C), 146.96, 149.12, 159.69 (d, *J* = 257.1 Hz, 1C); Purity (UV): 99%, t_R_: 5.11 min; MS (ESI+): *m/z* (%) = 259 (12), 258 (36), 257 (100) [M+H^+^], calc. 257.05.

*1-{5-[5-(pyridin-3-yl)thiophen-3-yl]pyridin-3-yl}ethan-1-one* **17**: yield: 39%, mp 166–167°C; IR (neat) 3079, 1419, 1124, 1023, 798, 695, 617 cm^−1^; ^1^H-NMR (300 MHz, CDCl_3_): *δ* (ppm) 2.68 (s, 3 H) 7.37 (ddd, *J* = 8.0, 4.9, 0.7 Hz, 1 H) 7.59–7.64 (m, 1 H) 7.64–7.68 (m, 1 H) 7.88–7.98 (m, 1 H) 8.39–8.46 (m, 1 H) 8.55 (dd, *J* = 4.8, 1.5 Hz, 1 H) 8.91 (d, *J* = 2.4 Hz, 1 H) 9.04 (d, *J* = 2.2 Hz, 1 H) 9.07 (d, *J* = 1.9 Hz, 1 H); ^13^C-NMR (75 MHz, CDCl_3_): δ (ppm) 26.85, 122.81, 122.85, 123.90, 129.93, 131.37, 132.35, 132.65, 133.41, 138.62, 142.06, 146.33, 148.19, 148.45, 150.76, 163.65, 196.41; Purity (UV): 98%, t_R_: 4.55 min; MS (ESI+): *m/z* (%) = 283 (7), 282 (18), 281 (100) [M+H^+^], calc. 281.07.

*5-[5-(pyridin-3-yl)thiophen-3-yl]pyridine-3-carboxamide* **19**: yield: 13%, mp 217–218°C; IR (neat) 3346, 1679, 1467, 1126, 1026, 802, 701, 617 cm^−1^; ^1^H NMR (300 MHz, Methanol-d_4_): *δ* (ppm) 9.10 (d, *J* = 2.24 Hz, 1H), 8.96 (d, *J* = 1.86 Hz, 1H), 8.93 (d, *J* = 1.68 Hz, 1H), 8.63 (t, *J* = 2.14 Hz, 1H), 8.50 (dd, *J* = 1.40, 4.94 Hz, 1H), 8.18 (ddd, *J* = 1.68, 2.61, 7.64 Hz, 1H), 8.07 (d, *J* = 1.49 Hz, 1H), 8.01 (d, *J* = 1.49 Hz, 1H), 7.52 (ddd, *J* = 0.84, 4.94, 8.01 Hz, 1H); ^13^C NMR (75 MHz, Methanol-d_4_) *δ* (ppm) 124.73, 124.75, 125.72, 131.54, 132.08, 132.87, 134.39, 135.11, 140.11, 142.85, 147.10, 148.07, 149.34, 150.42, 169.76; Purity(UV): 96%, t_R_: 5.47 min MS (ESI+): *m/z* (%) = 284 (14), 283 (38), 282 (100) [M+H^+^], calc. 282.06.

*3-[4-(1,3-thiazol-5-yl)thiophen-2-yl]pyridine* **29**: yield: 48%, mp 114–115°C; IR (neat) 3091, 1418, 1122, 1021, 798, 698, 600 cm^−1^; ^1^H-NMR (300 MHz, CDCl_3_): *δ* (ppm) 8.89 (dd, *J* = 0.65, 2.33 Hz, 1H), 8.69–8.76 (m, 1H), 8.55 (dd, *J* = 1.49, 4.84 Hz, 1H), 7.99–8.06 (m, 1H), 7.88 (ddd, *J* = 1.68, 2.42, 7.82 Hz, 1H), 7.50 (d, *J* = 1.49 Hz, 1H), 7.42 (d, *J* = 1.30 Hz, 1H), 7.33 (ddd, *J* = 0.84, 4.84, 7.92 Hz, 1H); ^13^C-NMR (75 MHz, CDCl_3_): δ (ppm) 122.06, 123.24, 123.69, 129.66, 132.73, 133.04, 133.48, 139.31, 141.77, 146.80, 148.94, 151.48; Purity(UV): 99%, t_R_: 4.72 min, MS (ESI+): *m/z* (%) = 247 (15), 246 (34), 245 (100) [M+H^+^], calc. 245.01.

*3-[4-(1-methyl-1H-pyrazol-4-yl)thiophen-2-yl]pyridine* **30**: yield: 21%, mp 119–120°C; IR (neat) 3103, 1420, 1129, 1023, 807, 703, 610 cm^−1^; ^1^H-NMR (300 MHz, CDCl_3_): *δ* (ppm) 3.94 (s, 3H), 7.23 (d, *J* = 1.1 Hz, 1H), 7.32 (dd, *J* = 8.0, 4.8 Hz, 1H), 7.43 (d, *J* = 1.1 Hz, 1H), 7.57 (s, 1H), 7.70 (s, 1H), 7.88 (dt, *J* = 8.0, 1.9 Hz, 1H), 8.52 (dd, *J* = 4.8, 1.4 Hz, 1H), 8.89 (d, *J* = 2.2 Hz, 1H); ^13^C-NMR (75 MHz, CDCl_3_): δ (ppm) 39.02, 118.23, 118.72, 123.22, 123.69, 126.99, 130.30, 133.00, 134.58, 136.93, 140.74, 146.67, 148.42; Purity(UV): 96.2%, t_R_: 4.10 min; MS (ESI+): *m/z* (%) = 244 (9), 243 (24), 242 (100) [M+H^+^], calc. 242.07.

*3-[5-(1,3-oxazol-5-yl)thiophen-3-yl]pyridine* **33**: yield: 18%, mp 86–87°C; IR (neat) 3075, 1415, 1129, 1027, 816, 706, 618 cm^−1^; ^1^H-NMR (300 MHz, CDCl_3_): *δ* (ppm) 7.28 (s, 1H), 7.31–7.39 (m, 1H), 7.47–7.52 (m, 1H), 7.58 (d, *J* = 1.2 Hz, 1H), 7.84–7.91 (m, 2H), 8.56 (dd, *J* = 5.2, 1.1 Hz, 1H), 8.87 (d, *J* = 2.2 Hz, 1H); ^13^C-NMR (75 MHz, CDCl_3_): *δ* (ppm) 121.55, 121.75, 123.11, 123.66, 130.83, 130.97, 133.45, 139.52, 146.48, 147.54, 148.69, 150.11; Purity(UV): 96%, t_R_: 3.69 min; MS (ESI+): *m/z* (%) = 231 (7), 230 (16), 229 (100) [M+H^+^], calc. 229.04.

*5-[4-(pyridin-3-yl)thiophen-2-yl]pyrimidine* **41**: yield: 47%, mp 173–174°C; IR (neat) 3045, 1416, 1104, 1027, 814, 708, 618 cm^−1^; ^1^H-NMR (300 MHz, CDCl_3_): *δ* (ppm) 9.16 (s, 1H), 9.00 (s, 2H), 8.90 (dd, *J* = 0.56, 2.24 Hz, 1H), 8.59 (dd, *J* = 1.49, 4.84 Hz, 1H), 7.92–7.92 (m, 1H), 7.90 (ddd, *J* = 1.68, 2.42, 8.20 Hz, 1H), 7.67 (d, *J* = 1.30 Hz, 1H), 7.62 (d, *J* = 1.30 Hz, 1H), 7.37 (ddd, *J* = 0.75, 4.84, 7.82 Hz, 1H), ^13^C-NMR (75 MHz, CDCl_3_): *δ* (ppm) 122.95, 123.70, 123.92, 128.26, 130.79, 133.48, 137.77, 140.36, 147.58, 148.86, 153.47 (2C), 157.66; Purity(UV): 98%, t_R_: 4.29 min; MS (ESI+): *m/z* (%) = 242 (5), 241 (15), 240 (100) [M+H^+^], calc. 240.05.

*3-[5-(1,3-thiazol-5-yl)thiophen-3-yl]pyridine* **48**: yield: 35%, mp 69–70°C; IR (neat) 3052. 1480, 1128, 1023, 769, 706, 612 cm^−1^; ^1^H-NMR (300 MHz, CDCl_3_): *δ* (ppm) 7.33–7.41 (m, 1 H) 7.45–7.50 (m, 2 H) 7.85–7.93 (m, 1 H) 8.03 (s, 1 H) 8.53–8.61 (m, 1 H) 8.72–8.78 (m, 1 H) 8.85–8.91 (m, 1 H); ^13^C-NMR (75 MHz, CDCl_3_): *δ* (ppm) 152.0, 148.4, 147.3, 139.7, 139.5, 134.4, 133.7, 132.0, 131.0, 124.5, 123.8, 121.6; Purity(UV): 99%, t_R_: 4.08 min, MS (ESI+): *m/z* (%) = 247 (10), 246 (16), 245 (100) [M+H^+^], calc. 245.01.

The synthetic procedures and analytical data of compounds **2**, **3**, **6, 7, 9–14, 16, 18, 21–24, 26–28, 31, 32, 34–40, 42–47** are given in File S2.

## Results and Discussion

### Identification of screening hits and lead generation

**Figure 1:**
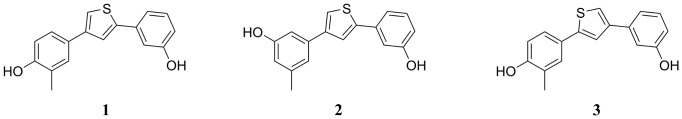
Structure of the hit compound 1 and two newly synthesized derivatives.

**Figure 2:**
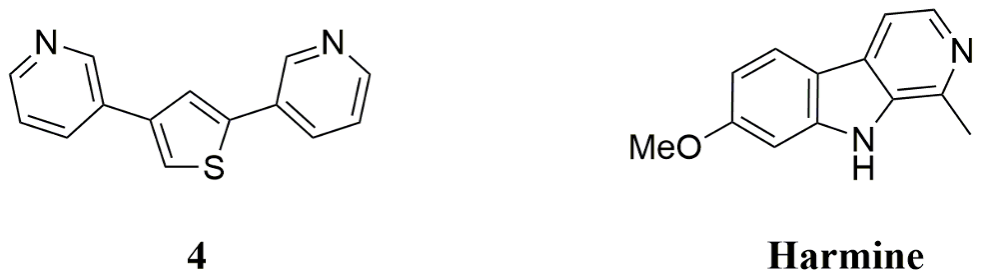
Structures of the Dyrk inhibitor 4 (this study) and harmine (originally isolated from *Peganum harmala*).

In order to discover novel inhibitors for the family of Dyrk kinases, we screened an in house library lacking any compounds with typical kinase inhibitor motifs at a concentration of 10 µM. During this campaign, hit compound **1** (Figure 1) was identified as a moderate inhibitor of Dyrk1A, exhibiting an IC_50_ of 2.0 µM. In order to assess the selectivity, a first counter screen was performed using casein kinase 2 (CK2α), a related kinase from the same CMGC family which was frequently shown to have overlapping active hits [81], [82]. Compound **1** proved to be rather inactive towards CK2α (15% inhibition at 5 µM). To explore first structure–activity relationships, we synthesized compound **2** as an analogue of compound **1** bearing both hydroxyl groups in the *meta*-position. This substitution pattern abolished the biological activity of the molecule (10% inhibition at 5 µM), suggesting that the positions of the hydroxyl groups might be crucial for hydrogen bond interactions. The constitutional isomer **3** of compound **1** showed comparable activity against Dyrk1A, but, interestingly, led to a significant loss of selectivity over CK2. This suggested that by altering the position of the sulfur atom in the thiophene core, it might be possible to influence the selectivity. We also tested 2,5-disubstituted thiophenes with similar phenolic substituents, but these compounds were all inactive (data not shown). The phenolic hydroxyl groups represented not only a weak spot for phase II metabolism, they were also responsible for the inhibitory activity of the hit compound against 17β-hydroxysteroid dehydrogenase 1 (HSD1), for which it had been originally designed [70]. In addition, hydrogen bond donor functions are negatively correlated with the ability to cross the blood–brain barrier [83], and a comparison of our hit compound with previously reported Dyrk inhibitors showed that hydrogen bond donor functions are not required for potent inhibition [49], [84]. Therefore, we aimed at replacing the hydrogen bond donor function of the hydroxyls by acceptor functions. Firstly, the methoxy–substituted analogue of compound **3** was tested which was available as a precursor compound. However, this derivative was completely inactive against Dyrk1A. A comparison with previously reported Dyrk inhibitors suggested that the distance of the hydrogen bond acceptor functions might be too large [49], [84]. Therefore, we decided to compact the bisphenol thiophene structure by including the hydrogen bond acceptor functions in aryl heterocycles, which logically led to 3-pyridyl rings. The resulting new compound class featured two hydrogen bond acceptor groups in a spatial distance comparable to that of the earlier described Dyrk1A inhibitor harmine (Table 1, Figure S1 in File S1). The prototype bispyridyl derivative **4** (Figure 2) turned out to be three times more potent towards Dyrk1A than the hit compound **1** (IC_50_ = 0.7 µM). Replacing one of the 3-pyridine rings by a 3-methoxy– substituted phenyl did not recover the biological activity, indicating again that the distance between the hydrogen bond acceptor functions is crucial for activity. In contrast to many known kinase inhibitor scaffolds, our novel lead compound **4** did not contain a tandem hydrogen bond donor/acceptor moiety, which could establish a strong affinity anchor with the highly conserved hinge region backbone, but on the other hand might compromise selectivity. Importantly, compound **4** did not affect the activity of CK2α, indicating that a decent degree of selectivity could be expected.

**Table 1:** Distances between the two hydrogen bond acceptor functions^a^.

Compound	Range of distances between H-bond acceptor functions [Å]
**1**	10.65–12.36
**4**	7.78–9.38
**Harmine**	7.85

a The energy was minimized using Chem3D Pro (Version 13.0.0.3015; Method: MM2). The ranges denote the distances measured in different conformers of similar energy.

It seemed straightforward to investigate whether the binding affinity of **4** could be increased by optimization of both the position and the strength of the two hydrogen bond acceptor functions. In addition, already small differences in the angle and distance between these functions might translate into significant binding preferences for the one versus the other Dyrk isoform. Furthermore, independent modulation of the electrostatic potential as well as the steric properties of the exterior ring systems provided another means to optimize the selectivity profile.

### Chemistry

A diverse set of aromatic azaheterocycles was attached to the 2- and 4-positions of the thiophene core. Since the compounds were conveniently accessible by consecutive Suzuki cross coupling reactions [85], it was possible to adapt the synthesis to an automated robotic system for diversification purposes. A series of N-heteroaryl bromides or boronic acids was selected, including pyridine isomers, substituted pyridines, pyrimidines, pyridinone, five-membered heterocycles e.g. pyrazoles, thiazoles, oxazoles and fused heterocycles, with molecular masses below 140 g/mol, so that the final compounds would not exceed a mass of 300 g/mol. All building blocks were commercially available for prices below US$ 50 per gram.

**Figure 3:**
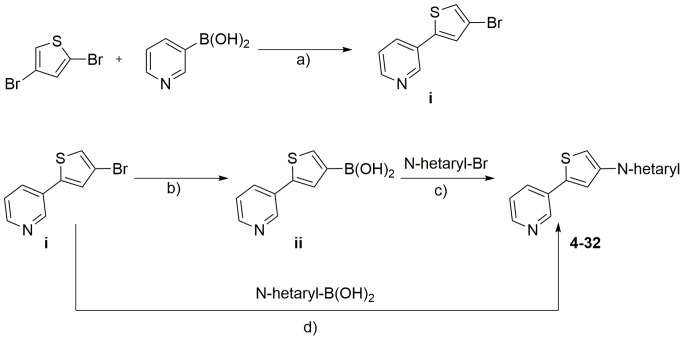
Synthesis of target compounds 4 to 32*^a^*. *^a^*Reagents and conditions: a) Na_2_CO_3_, Pd(PPh_3_)_4_, Dioxane/Water, Reflux, 15 hours; b) (iPrO)_3_B, nBuLi, Toluene/THF, −78°C, 75 min, 2 M HCl, −25°C, 30 min; c) N-hetaryl bromide, CS_2_CO_3_, Pd(dppf)Cl_2_, DMF/Water, Reflux, 15 hours; d) N-hetaryl boronic acid, Cs_2_CO_3_, Pd(dppf)Cl_2_, DMF/Water, Reflux, 15 hours.

**Figure 4:**
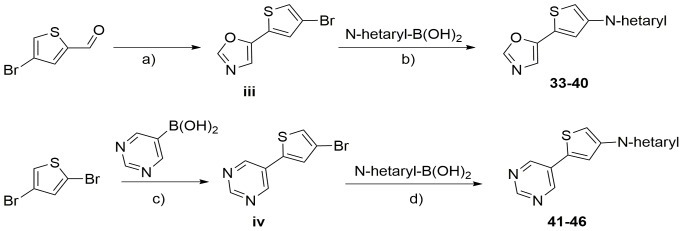
Synthesis of target compounds 33 to 46*^a^*. *^a^*Reagents and conditions: a) Tosylmethylisocyanate, Na_2_CO_3_, Methanol, Reflux, 4 hours; b) Cs_2_CO_3_, Pd(dppf)Cl_2_, DMF/Water; Reflux, 15 hours; c) Na_2_CO_3_, Pd(PPh_3_)_4_, Dioxane/Water, 90°C, 48 hours; d) Cs_2_CO_3_, Pd(dppf)Cl_2_, DMF/Water; Reflux, 15 hours.

**Figure 5:**
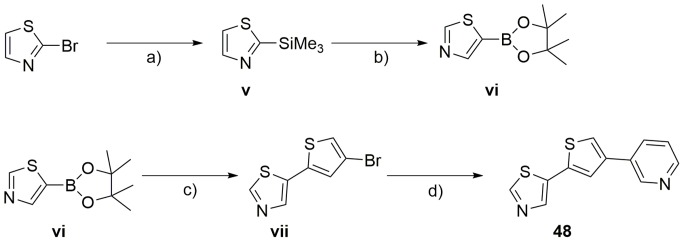
Synthesis of Compound 48*^a^*. *^a^*Reagents and conditions: a) nBuLi, diethyl ether, −78°C, 60 min; trimethylsilylchloride, −78°C, 60 min; b) nBuLi, THF, −78°C, 35 min; (*i*PrO)_3_B, −78°C, 90 min; pinacol, RT, 10 min; c) Dibromothiophene, Na_2_CO_3_, Pd(PPh_3_)_4_, Dioxan/Water, 90°C; d) 3-Pyridylboronic acid, Cs_2_CO_3_, Pd(dppf)Cl_2_, DMF, Reflux.

Starting from compound **4**, we synthesized three compound series. Within the first series we kept the 3-pyridyl moiety in the 2-position of the thiophene core constant and varied the substituent in 4-position of the central core (compounds **5** to **32**). Figure 3 outlines the synthetic route for compounds **5** to **32**. 2,4-Dibromothiophene was reacted with 3-pyridylboronic acid to yield (**i**). Compound (**i**) was either directly reacted with an appropriate heteroaryl boronic acid or first converted to the corresponding boronic acid (**ii**) and subsequently reacted with an appropriate heteroaryl bromide to yield compounds **5** to **32**. Figure 4 illustrates the synthesis of compound series 2 (compounds **33** to **40**) and 3 (compounds **41** to **46**). Series 2 was furnished with an oxazole moiety in 2-position of the thiophene core, which could be easily introduced by reaction of 4-bromo-2-thiophenecarbaldehyde with Toluenesulfonylmethyl isocyanide (TosMIC). The resulting precursor (**iii**) was directly used for the synthesis of compounds **33** to **40** by a subsequent Suzuki cross coupling reaction. In series 3, a pyrimidine moiety was introduced in 2-position of the central thiophene core by reaction of 5-pyrimidine boronic acid with 2,4-dibromothiophene. The resulting intermediate (**iv**) was used in a subsequent Suzuki cross coupling to yield compounds **41** to **46**. In addition to the compound series named above, we synthesized an additional molecule, bearing the 3-pyridyl moiety in 4-position of the thiophene core (Figure 5). The resulting compound **48** represented an isomer of compound **29**, where the relative position of the sulfur atom is moved by one atom. Therefore, 2-bromo-thiazole was reacted with trimethylsilyl chloride to yield (**v**), which was converted to the corresponding boronic ester (**vi**). Suzuki cross coupling of (**vi**) with 2,4-dibromothiophene yielded (**vii**), which was reacted with 3-pyridyl boronic acid to compound **48**.

### Biological activity

Compound **4** is directed to the ATP-binding site. Because the synthesized compounds have not been described in the context of kinase inhibition yet, we first investigated the potential mechanism of inhibition. Given that the two hydrogen bond acceptor functions in **4** were superimposing with those in harmine (cf. Figure S1 in File S1), it was most likely that the compounds would also target the ATP-binding site, although they did not exhibit a fully coplanar shape. In a kinetic experiment, we were able to confirm that Dyrk1A was inhibited by **4** in a clearly ATP-competitive manner (Figure S2 in File S1). Hence, for the discussion of the structure–activity relationships (SAR), it was assumed that the compounds were addressing the ATP-binding pocket.

### Structure-activity relationships of the 2,4-bisheterocyclic substituted thiophenes

Starting from **4**, we first investigated the influence of different heterocycles in the 4-position of the central thiophene on the biological activity while the 3-pyridyl in 2-position was preserved. The biological activities of this first compound series are shown in Table 2, with the most potent compounds being marked by asterisks. Firstly we probed the impact of an additional methyl group in the variable part of the compound. Compounds **5** to **7** featured 3-pyridyl moieties bearing a methyl substituent at different positions. Compound **5** maintained approximately the activity towards Dyrk2, whereas a slight loss of activity was observed towards Dyrk1A and 1B. The selectivity against CK2 was not affected. For compound **6** and **7**, we observed a significant loss of activity towards the Dyrk kinases. In the case of **6**, the sixfold decrease in affinity is probably caused by the methyl group in *ortho*-position to the thiophene linkage. This presumably forced the pyridine ring to rotate out of plane which was not tolerated by the binding pocket. In compound **7**, the *para*-methyl presumably caused a steric clash within the ATP pocket, thus preventing the compound from assuming an optimal binding position. The impact of the *para*-methyl group was less pronounced with Dyrk2 and resulted only in a twofold loss in activity. We also introduced an additional hydrogen bond acceptor function in *meta*-position to the nitrogen (**8**). This modification enhanced the activity towards Dyrk1B and Dyrk2, whereas the potency towards Dyrk1A was similar to that of **4**. A sterically more demanding, flexible residue like an ethoxy group (**10**) was not tolerated at this position. Similarly to **7**, the spatial requirements of the methoxy group in the *para*-position to the thiophene linkage (**9**) led to a complete loss of activity towards the Dyrk isoforms. Apparently, the electron density at the pyridine nitrogen could not be efficiently enhanced, because the electron donating substituents were not tolerated at the relevant position. To further investigate the optimal relative position of the pyridyl nitrogen, we introduced the 4-pyridyl at one side, resulting in compounds **11** and **12**. This completely abolished the biological activity, confirming that the position of and distance between the hydrogen bond acceptor functions was critical to the activity. The next step was to switch from electron–donating substituents to electron–withdrawing substituents in the 3-pyridyl ring. To this end, we introduced nicotinonitrile, 3-trifluoromethylpyridyl, 3-fluoropyridyl, 3-chloropyridyl, 3-acetylpyridyl, nicotinic acid methyl ester, and nicotinamide as thiophene-4-substituents (**13**–**19**). As a result, we found that the nitrile and the trifluoromethyl group were not tolerated well in all tested Dyrk kinases, whereas fluorine and chlorine affected the biological activity only slightly compared with compound **4**, thus offering a potential option to increase the metabolic stability. None of the substitutions provoked CK2 inhibition, indicating that the scaffold – though being small – did not bind to the ATP-binding pocket of this related kinase. We also introduced a quinoline (**20**) and an isoquinoline moiety (**21**) into the 4-position of the thiophene to enhance the hydrophobicity and extend the aromatic system. The quinoline derivative was slightly more active than the isoquinoline, which was probably related to the different spatial orientation of the additional hydrophobic π-system (Figure S3 in File S1). We assumed that both moieties protrude into different areas of the ATP pocket. However, they probably prevented an optimal hydrogen bond interaction so that the potency was actually reduced compared with **4**. We also examined the influence of an additional hydrogen bond acceptor function by synthesis of a pyrimidine–substituted thiophene **22**. This modification decreased the potency toward the target kinases, which may have been caused by an inappropriate electrostatic potential and/or attenuated hydrogen bond acceptor strength of the nitrogen involved in hydrogen bonding [86]. On the other hand, the positional isomer **41** (see further below) restored the activity towards Dyrk1A, however with a two times lower potency towards Dyrk2. Hence, the regioisomeric pair of compounds **22** and **41** provided another example that the relative position of the sulfur in the thiophene core had an influence on the potency and selectivity.

**Table 2:** Biological activity of 3-(thiophen-2-yl)pyridine derivatives with diversification at the 4-position of the thiophene core.

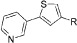					
Compound	Structure	IC_50_ [µM]^a^			
		Dyrk1A	Dyrk1B	Dyrk2	CK2α
4*	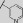	0.7	0.7	0.7	18.5%
5*	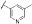	0.9	1.2	0.6	5%
**6**	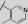	4.4	8.0	10% (1 µM)	0%
**7**	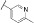	4.7	3.4	1.6	0%
8*	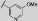	0.7	0.3	0.2	16%
**9**	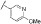	36	34	No inhibition	0%
**10**	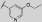	45%	6% (1 µM)	22% (1 µM)	0%
**11**	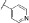	10%	10%	20%	0%
**12**	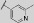	23%	No inhibition	37.5%	n.d.
**13**	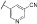	2.6	30%	44%	3%
**14**	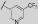	3.1	5.4	3	0%
15*	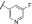	0.9	1.4	0.4	0%
**16**	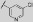	1.2	1.9	0.7	14%
17*	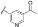	0.9	1.1	0.9	0%
**18**	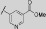	1.2	1.7	1.5	14%
19*	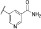	0.9	1.5	0.3	4.2%
**20**	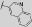	1.1	0.9	1.8	27%
**21**	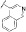	3.5	3.2	2.9	6%
**22**	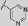	1.8	3.5	1.8	0%
**23**	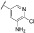	1.3	1.4	0.3	0%
**24**	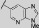	37%	13% (1 µM)	0.6	7%
**25**	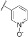	4%	n.d.	n.d.	n.d.
**26**	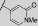	No inhibition	No inhibition	27%	n.d.
**27**	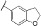	No inhibition	No inhibition	31%	n.d.
**28**	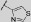	31%	n.d.	6.7	n.d.
29*	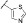	0.1	0.1	0.4	0%
30*	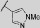	0.3	0.4	0.2	9.5%
**31**	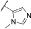	44%	9.1	5.1	n.d.
**32**	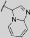	2.2	0.6	0.4	0%

a Given are mean values of at least two independent experiments, S.D. <10%. Compounds exhibiting an IC_50_ value below 1 µM towards Dyrk1A are marked by an asterisk. Percent values indicate the percentage of inhibition at 5 µM if not stated otherwise (other test concentrations are indicated in brackets).

A further extension of the pyridine in 4-position by disubstitution (**23**) or by annelation (**24**) significantly enhanced the selectivity for Dyrk2. Within the three screened Dyrk family members, compound **24** was specific for Dyrk2.

Compounds **25** to **27** were completely inactive against the Dyrk kinases. Since we found that the introduction and modification of a six-membered ring systems did not achieve a significant increase in potency towards Dyrk1A and Dyrk1B, we decided to introduce five-membered ring systems in the 4-position of the thiophene core. While the thiazole derivative **28**, with the nitrogen being located in 2-position relative to the thiophene linkage, was inactive against Dyrk1A/B and weakly inhibited Dyrk2 (IC_50_ = 6.7 µM), the positional isomer **29** turned out to be a potent inhibitor of all Dyrk kinases with a preference for Dyrk1A/B. When tested in parallel in our assay, the IC_50_ values for **29** and the reference compound harmine were almost identical. The 5 to 7-fold enhancement of the potency (towards Dyrk1A and 1B, respectively) compared with the pyridine analogue **4** was remarkable, given the fact that the hydrogen bond acceptor strength of the thiazole is weaker than that of the pyridine [86]. Probably the angle of the nitrogen toward the hydrogen bond donor is more favorable than with the pyridine, thus compensating for its reduced acceptor strength.

Introduction of a methyl pyrazole also yielded one of the most potent inhibitors of this series (**30**). However, the selectivity switched to Dyrk2. In contrast, compound **31**, bearing a methyl imidazole moiety with the methyl group in *ortho*-position to the thiophene linkage, displayed a largely reduced activity against all Dyrk kinases. The methyl group probably forced the ring to rotate out of plane, thus abolishing the favorable co-planar conformation, similar to compound **6**. Extension of the favorable imidazole ring to the imidazo[1,2-*a*]pyridine produced compound **32** with a distinct selectivity profile, showing good inhibition of Dyrk1B and Dyrk2 but significantly reduced activity toward Dyrk1A.

In parallel to the compounds from series 1 (Table 2), we synthesized two additional compound classes (Tables 3 and 4). The pyridine in 2-position was replaced by oxazole (Table 3) and by pyrimidine (Table 4), respectively, while the 4-position was varied by a set of different heterocycles. Within these series we identified four Dyrk inhibitors with IC_50_ values around 1 µM. Compound **33** and **41** were equally active towards Dyrk1A. However, the pyrimidine–substituted congener **41** displayed a superior selectivity (cf. discussion of the matched isomer **22** above). Interestingly, in this series, the introduction of a methyl group in *meta*-position to the thiophene connection of the pyridine substituent (**43**) decreased the biological activity as well as the selectivity profile within the Dyrk family. Introduction of a chlorine substituent also abolished the activity (**34** and **42**). These substituent effects are in sharp contrast to those seen with the bis-pyridyl derivatives **5** and **16**, demonstrating that they are specifically dependent on the type of the partner heterocycle at the other thiophene position.

**Table 3:** Biological activity of 2-(thiophen-2-yl)oxazole derivatives with diversification at the 4-position of the thiophene core.

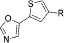					
Name	R	IC_50_ [µM]^a^			
		Dyrk1A	Dyrk1B	Dyrk2	CK2α
33*	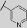	0.8	1	1.34	0%
**34**	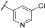	0%	18%	12%	-
**35**	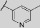	1.2	1.1	1.5	−15%
**36**	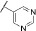	25%	40%	16%	-
**37**	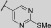	7%	21%	5%	-
**38**	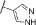	1.5	2.2	1.5	−20%
**39**	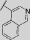	20%	8.07	19%	-
**40**	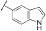	18%	9%	30%	-

a See footnote of Table 2.

**Table 4:** Biological activity of 5-(thiophen-2-yl)pyrimidine derivatives with diversification at the 4-position of the thiophene core.

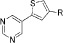					
Name	R	IC_50_ [µM]^a^			
		Dyrk1A	Dyrk1B	Dyrk2	CK2α
41*	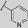	0.9	1.8	3.5	0%
**42**	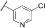	1%	5%	0%	-
**43**	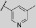	5	2.2	3.8	−15%
**44**	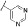	2%	27%	27%	-
**45**	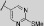	8%	20%	−24%	-
**46**	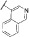	9%	33%	15%	-

a See footnote of Table 2.

Notably, none of the oxazole derivatives reached the potency range of the thiazole congeners **29** and **48** (see below), even though both heterocycles exhibit a comparable hydrogen bond acceptor strength [86]. However, compared with the oxazole ring system, the thiazole ring displays a higher aromatic character as a major difference. The Bird Index (BI), a measure for aromaticity relative to benzene (BI 100), has a value of 79 for thiazole, which is closer to pyridine (BI 86) than that of oxazole (BI 47) [87]. In consequence, the π–electrons are less delocalized in the oxazole ring but more confined to the double bonds, thus decreasing potential surface for CH–π interactions with the aliphatic side chains of the ATP- binding pocket in Dyrk1A/B. Interestingly, though, we found later that the oxazole compound **33** was still accepted by the Clk1 ATP-binding site (see below), conferring some selectivity for the latter kinase. In contrast, the thiazole congeners **29** and **48** turned out to be dual inhibitors of Dyrks and Clk1/4 (see under “selectivity profile”).

Inspired by the distinct results obtained with the isomeric pair **22** and **41**, we also synthesized isomers of **21** and **29** in which the relative position of the sulfur atom in the thiophene core was changed. This modification led to compound **47** and **48**, respectively (Table 5). Interestingly, the activity of **48** towards Dyrk1A and Dyrk1B was not affected compared to **29**, but the Dyrk2 activity was increased tenfold. Compound **47** was slightly more potent than the first isomer **21**, but without major changes in the selectivity profile.

**Table 5:** Biological activities of compounds 47 and 48 possessing a changed relative position of the thiophene sulfur compared to their regioisomers 21 and 29.

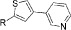					
Compound	Structure	IC_50_ [µM]^a^			
		Dyrk1A	Dyrk1B	Dyrk2	CK2α
**47**	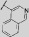	1.5	2.4	1.2	3%
48*	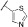	0.1	0.07	0.04	0%

a See footnote of Table 2.

Altogether, our results suggested that depending on the size and type of the heterocycles, the thiophene scaffold allowed modulation of both selectivity and potency simply by interchanging the positions of the thiophene substituents.

Thiazole was found to generate potent compounds both at the 2- and the 4-position of the thiophene core in the disubstituted inhibitors. Expectedly, high ligand efficiencies were calculated for both thiazole compounds **29** and **48** (Table S1 in File S1). According to these results, the design of future refined libraries of Dyrk inhibitors might include a thiazole ring in combination with other azaheterocycles.

### Evaluation of the Drug-like properties

The major indication for Dyrk1A inhibitors are neurodegenerative diseases such as Alzheimers disease [4], [5], [9], [10]. Thus, it is prerequisite for those inhibitors to cross the blood–brain barrier. In the literature, several physicochemical properties of CNS–active drugs and lead compounds have been analyzed, thus providing parameters to estimate the probability of a brain penetration [83]. To this end, we experimentally determined the physicochemical properties of compound **4** including logP, pk_a_ and logS. The obtained data were then compared with the corresponding calculated physicochemical properties (Table 6). Since the predicted parameters were in a good agreement with the experimental data for the prototype compound **4**, we decided to calculate the most important predictive parameters of our best compounds and compared them to those of the CNS–active inhibitor harmine (Table S2 in File S1). Almost all calculated parameters showed ideal values, with the number of hydrogen bond donors being even more favorable than in the case of harmine. Only the calculation of the size of the polar surface area (PSA) [88] raised the question as to whether the thiophene sulfur should be included or not. However, since the calculation of the molecular electrostatic potential of each compound revealed no particular polarity for the aromatic sulfur atom (data not shown), it might be correct to exclude the sulfur from the calculation, however both values are given in Table S2 in File S1. Altogether, the probability of CNS penetration was predicted to be high.

**Table 6:** Physicochemical properties of compound 4.

Physicochemical property	Experimental	calculated
logP	2.47	2.61^a^
pk_a1_	3.48	4.23^a^
pk_a2_	4.8	4.86^a^
logS	−2.67	−2.81^b^
Solubility mg/ml	0.50	0.37^b^

a Calculation was performed using JChem for Excel (Version 6.0, 2013, ChemAxon Ltd., www.ChemAxon.com);

b Calculation was performed using ACD/Percepta 2012 (ACD/Labs).

### Evaluation of metabolic stability

In addition to the assessment of the physicochemical parameters, the most potent lead compounds **29**, **30** and **48** were evaluated for their phase I metabolic stability using rat liver microsomes. Samples were taken at defined time points, and the remaining percentage of parent compound was determined by LC-MS/MS. Half-life and intrinsic clearance were calculated and compared to the two reference compounds diazepam and diphenhydramine, but also to harmine (Table 7). All of the new lead compounds but not harmine showed significantly longer half-lives than the antihistaminic drug diphenhydramine. In particular, compound **30** was considerably more stable than harmine and showed a good half-life and predicted clearance ranging between those obtained for the drugs diphenhydramine and diazepam. The two isomeric compounds **29** and **48** displayed a similar metabolic stability and were slightly more stable than harmine.

**Table 7:** Metabolic stability of compounds 29, 30 and 48 and reference compounds against rat liver microsomes^a^.

Compound	Half-life [min]	Cl_blood_ b [mL/min/kg]
Harmine	18	53
29	27	49
30	53	34
48	31	47
Diphenhydramine	17	53
Diazepam	96	25

a 0.225 mg/mL protein, NADP^+^-regenerating system, [inhibitor]: 0.5 µM, incubation at 37°C, samples taken at 0, 15, 30, and 60, 90 min, determination of parent compound by MS.

b Cl_blood:_ estimated blood clearance in rats as calculated based on *in vitro* intrinsic clearance. The values are representative for two independent experiments that essentially gave the same results.

### Cell-based functional and toxicity assays

**Figure 6:**
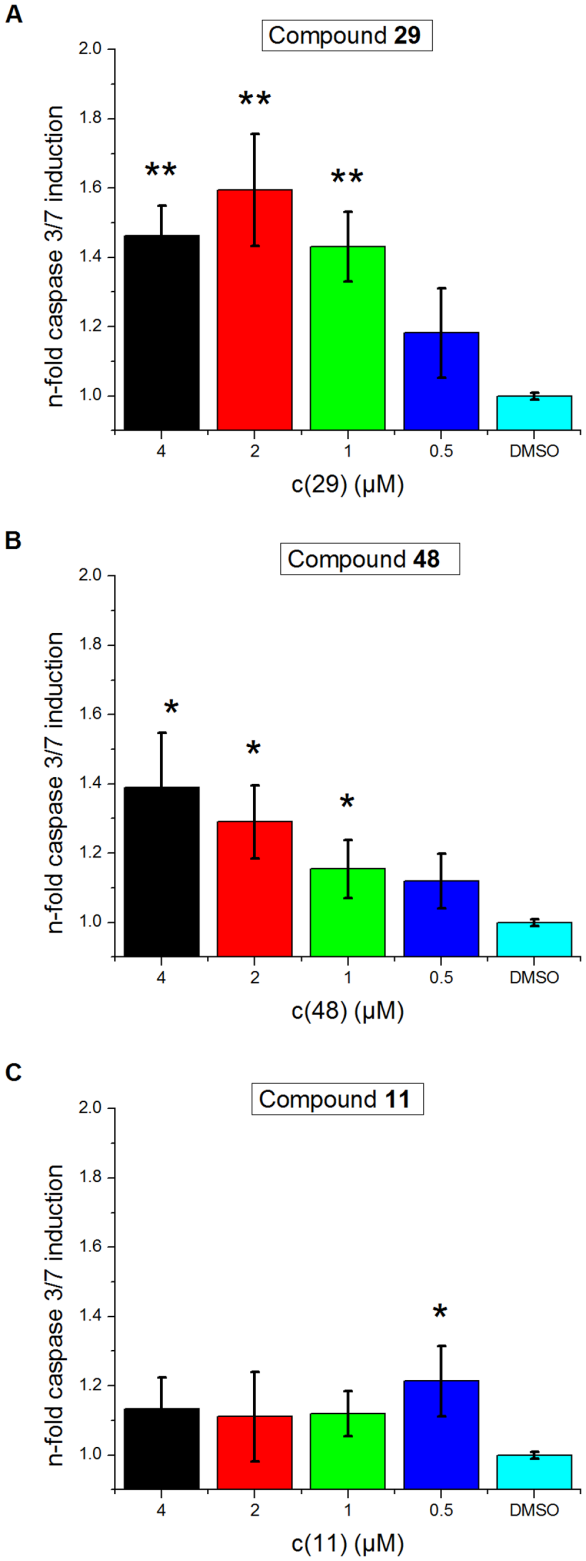
The Dyrk1B inhibitors 29 and 48 induce Caspase 3/7 activity in U2OS cells. U2OS cells were incubated overnight with the indicated concentrations of the active inhibitors **29** (A) and **48** (B), which resulted in a concentration–dependent increase of Caspase-3/7 activity. In contrast, incubation with the inactive congener **11** (C) slightly enhanced the background caspase activity independent of the compound concentration. Values from one out of three separate experiments are shown that gave essentially similar results. The standard deviation is given as y–error bar. One asterisk indicates significance with a p value <0.05, two asterisks indicate significance p<0.01.

Having observed a potent inhibition of Dyrk1B, and because Dyrk1B inhibitors which are more potent than harmine had barely been reported, we performed different assays to evaluate the ability of our compounds to inhibit Dyrk1B in cells. It was previously shown that the inhibition of Dyrk1B in tumor cell lines in which this kinase is overexpressed triggers the activation of the caspase signaling cascade resulting in apoptosis [89], and causes an increased production of reactive oxygen species (ROS) also contributing to cell destruction [90]. For our experiments, we chose the U2OS sarcoma cell line as a model, in which a strong overexpression of Dyrk1B had been demonstrated [89]. The cells were starved and incubated with the most potent inhibitors from our series and in addition with one inactive congener (**11**) as a further control. The activity of caspase-3/7 was measured as an indicator of apoptosis induction after 48 hours. We found that compounds **29** and **48**, which had inhibited purified Dyrk1B with IC_50_ values of 100 and 70 nM, respectively, triggered a clear increase of the caspase-3/7 activity in a concentration–dependent manner (Figures 6A and 6B). Notably, the induction of caspase-3/7 by **29** was observed already at a concentration of 0.5 µM, although the values at this concentration failed to reach statistical significance. In contrast, the Dyrk1B–inactive analogue **11** caused only a slight increase of the caspase-3/7 activity compared with the DMSO control, which however, did not show a concentration–dependency, suggesting that it was a non-specific effect (Figure 6C). The effects observed with **29** and **48** were fully consistent with an inhibition of Dyrk1B in this tumor cell line, which was previously reported to respond by an increased apoptosis rate after down-regulation of Dyrk1B by siRNA [89].

**Figure 7:**
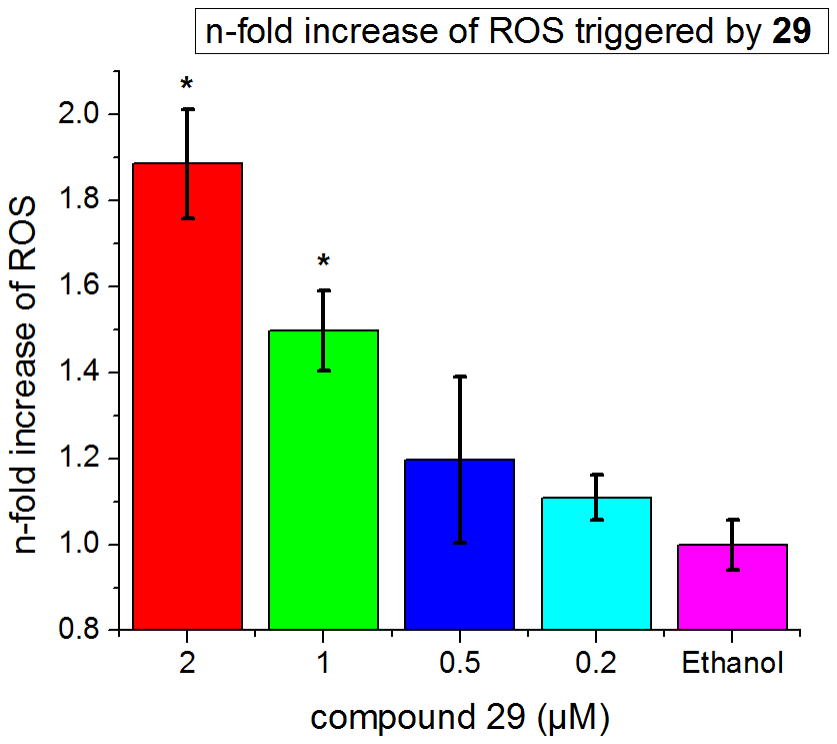
The Dyrk1B inhibitor 29 triggers the production of reactive oxygen species in U2OS cells. Incubation of U2OS cells with **29** caused an increased intracellular conversion of didydroethidium to the stronger fluorescent ethidium, indicating raised levels of reactive oxygen species compared with the ethanol control. Data shown are representative of two independent experiments. Asterisks indicate statistical significance (p value <0.05).

Another known biological consequence of Dyrk1B inhibition in tumor cells is the increased generation of ROS [90]. The ability of our most potent compound **29** to trigger this response in U2OS cells was tested using the cell permeable dihydroethidium as an indicator dye. In the presence especially of the superoxide anion radical (O_2_·^−^), this dye is oxidized to 2-hydroxyethidium, giving rise to increased fluorescence after intercalating with the cellular DNA [91]. Indeed, **29** considerably enhanced the fluorescence of the reporter dye in a concentration–dependent manner, already starting at 0.2 µM (Figure 7). This result was indicative of an increased production of ROS as a consequence of Dyrk1B inhibition.

To provide further evidence that Dyrk1B was the intracellular target of our compounds, we analyzed by Real-Time PCR whether they would modulate some major Dyrk1B–regulated gene expressions in the expected manner. To this end, we selected a comprehensive set of genes representing the distinct Dyrk1B–dependent gene regulatory pathways that were previously reported in literature. For instance, Dyrk1B phosphorylates the class II histone deacetylase (HDAC)5, leading to a reduced nuclear accumulation and suspended biological function of this epigenetic modifier, thus ensuing an increased transcription of a number of genes [92], [93]. We selected CDH4 (cadherine-4) and FGF2 (basic fibroblast growth factor), whose transcription was found to be clearly suppressed by HDAC5 activity in a large microarray analysis [66]. Treatment of U2OS cells with our Dyrk1B inhibitors was therefore expected to induce a down-regulation of these genes due to increased migration of HDAC5 to the nucleus. Indeed, both **29** and **48** caused a slight reduction of the mRNA expression, which was somewhat more pronounced with FGF2 (Table 8).

**Table 8:** Modulation of Dyrk1B–regulated gene expressions by compounds 29 and 48 as analyzed by Real-Time PCR^a^.

Target gene mRNA	29	29	48	48
	5 µM	10 µM	5 µM	10 µM
CDH4	−1,4±0.2	−1,5±0.3	−1,4±0.1	−1,5±0.2
FGF2	−1,4±0.1	−1,8±0.2	−1,7±0.1	−1,7±0.3
BIM	2,4±0.2	2,5±0.4	2,7±0.3	2,6±0.5
TRADD	2,5±0.3	3,0±0.3	1,2±0.2	3,0±0.6
FasL	1,2±0.1	2,7±0.4	1,2±0.1	1,9±0.3
SOD2	−2,3±0.2	−2,3±0.3	−2,4±0.1	−2,1±0.4
CP	−2,7±0.3	−2,9±0.6	−2,0±0.3	−2,2±0.4

a Values indicate fold up-regulation (positive numbers) or down-regulation (negative numbers) of mRNA expression, relative to DMSO–treated cells. Given are mean values of at least two independent experiments, n = 3, ±S.D.

Furthermore, Dyrk1B was implicated in the down-regulation of the FoxO family of transcription factors; siRNA–mediated knockdown of Dyrk1B in ovarian cancer cells resulted in apoptosis accompanied by nuclear translocation of FoxO1 and/or FoxO3A as well as increased BIM (BH3-only member of the BCL-2 family) and TRADD (Tumor necrosis factor receptor type 1–associated DEATH domain protein) expression, as well as caspase-3 and PARP cleavage [94]. In addition, it was reported that Dyrk1A co-localizes with FoxO and may phosphorylate FKHR (Forkhead in rhabdomyosarcoma) at Ser329, decreasing the ability of FKHR to stimulate gene transactivation and reducing its nuclear localization. In agreement, treatment of fibroblasts with the Dyrk1A/B inhibitor harmine had resulted in elevated FoxO-DNA binding activity and increased nuclear accumulation [95]. Hence, the effect of our inhibitors on FoxO signaling was monitored by analyzing the expressions of the transcriptional targets BIM, TRADD and FasL (Fas ligand). In full accordance with the mentioned reports, incubation of U2OS cells with our inhibitors resulted in an increased expression of all three pro-apoptotic genes (Table 8), although a concentration–dependency was not clearly seen in the case of the BIM expression. However, it is possible that BIM was already maximally transactivated at the lowest concentration of each inhibitor tested.

Altogether, using the complementary approach of apoptotic gene expression analysis, we were able to confirm that the compounds induce apoptosis, in agreement with the activation of caspase 3/7 (Figure 6). According to previous findings, the release of FoxO repression by pharmacological inhibition of Dyrk1B might be the responsible pathway, at least partially. In the light of the potentially synergistic roles of Dyrk1A and Dyrk1B in the suppression of FoxO function, dual inhibitors of both Dyrk isoforms, such as those presented here, might be even more effective.

In addition, Dyrk1B was described to increase the expression of antioxidant enzymes by an as yet unidentified pathway. Specifically, it was reported that shRNA–induced silencing of Dyrk1B led to a reduced mRNA expression of the antioxidant genes superoxide dismutase (SOD)2 and ferroxidase (also called ceruloplasmin, CP) in pancreatic cancer cell lines, accompanied by an increase in ROS levels [90]. As can be seen from Table 8, the mRNA expression of both SOD2 and CP was reduced more than twofold in the presence of the compounds compared to the DMSO control. Like in the case of BIM, there was only a weak concentration dependency – if at all – at the concentrations chosen for the experiment. Again, a possible explanation could be that the maximum suppression of transcription might already have been reached at 5 µM. This is supported by the fact that a slight increase of ROS production was already observed at 0.2 µM of compound **29** (Figure 7).

Collectively, the results of our transcription profiling, obtained with seven different genes from three different Dyrk1B–regulated pathways, were in full agreement with the previous literature reports. Moreover, the profiling data were consistent with the results from our biochemical assays regarding Caspase3/7 activation (Figure 6) and ROS production (Figure 7). Hence the transcription profiling provided strong evidence that compounds **29** and **48** indeed targeted Dyrk1B in the U2OS cells.

**Figure 8:**
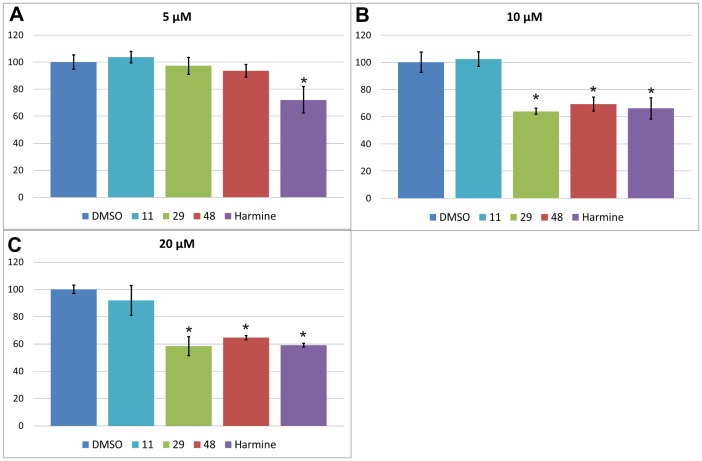
Influence of the Dyrk inhibitors 29 and 48 at three different concentrations (A–C) on the cell growth of V79 hamster lung fibroblasts in comparison to harmine. Compound **11** is of the same chemotype as **29** and **48** but was inactive with respect to Dyrk/Clk inhibition. Intracellular ATP levels were quantified as a measure for cell viability and proliferation rates. Results of one out of three independent experiments are presented, which all showed the same tendency. The standard deviation is indicated as y-error bars. Asterisks indicate statistical significance (p value <0.05).

To further rule out a general cytotoxicity of the compounds, we tested the potential effects on cell growth of V79 hamster lung fibroblasts as a model for non-tumor cells which do not depend on overexpressed Dyrks. Cellular ATP levels were determined as a measure for cell viability and proliferation using a luminescence–coupled assay. As shown in Figure 8, harmine appeared to be slightly toxic already at 5 µM, whereas compounds **29** and **48** showed no influence on cell viability at this concentration. However, there was no significant difference anymore at the higher concentrations (10 and 20 µM). Thus, all active dual Dyrk/Clk inhibitors seemed to affect cell growth, whereas a control compound (**11**) of the same chemotype but without effect on Dyrk kinases (in the cell-free assays) showed no influence. We thus concluded that at least part of the effect shown by all Dyrk/Clk inhibitors at higher concentrations might be mechanism–related. One possible explanation might be that mitochondrial ATP production was slightly reduced at higher concentrations of the compounds due to the increased ROS generation as a consequence of SOD2 down-regulation (see also above) [96].

### Binding model

**Figure 9:**
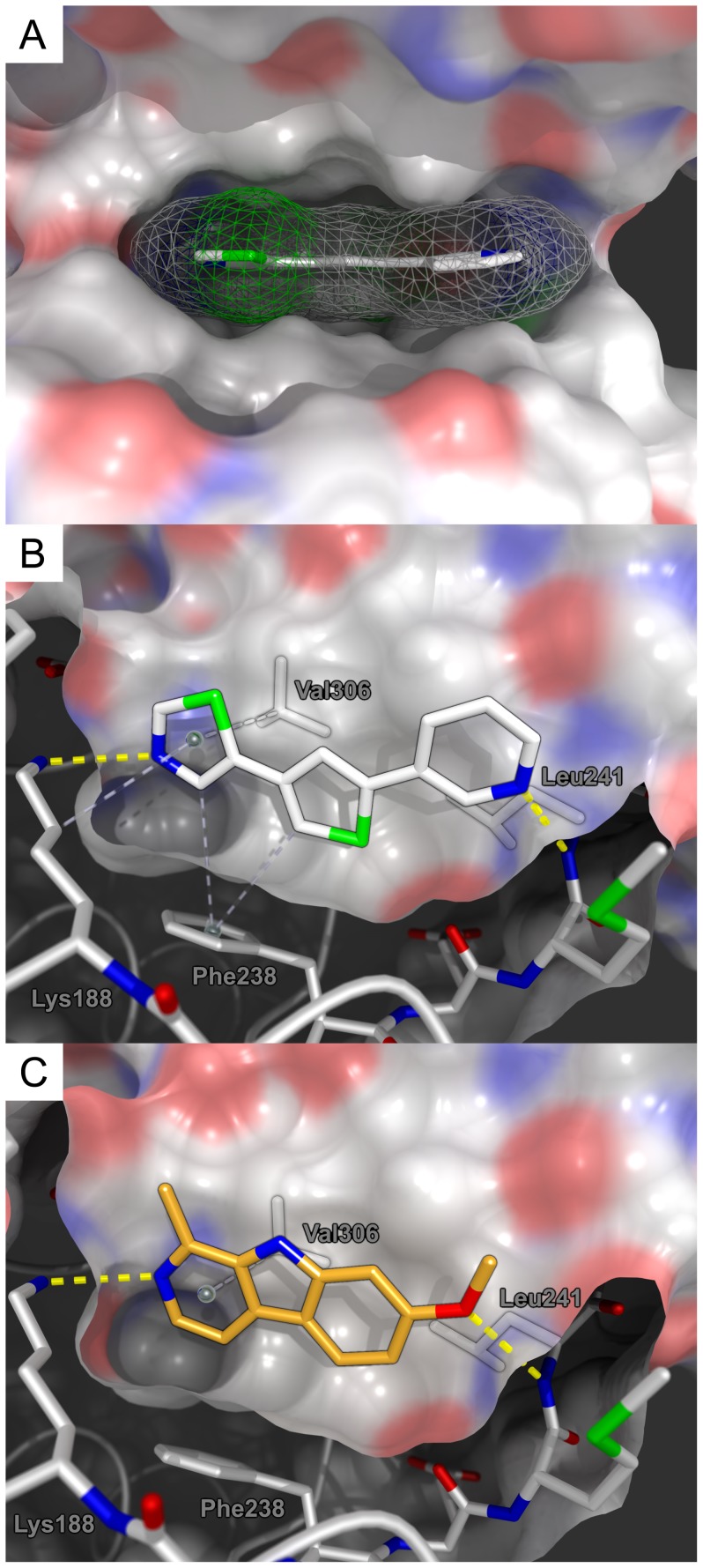
Predicted structure of the inhibitor-enzyme complex of 29 and Dyrk1A (A and B) in comparison with the corresponding cocrystal structure of harmine (C; PDB accession code: 3ANR). (A) Spatial orientation and space-filling (wireframe) of compound **29** within the ATP pocket of Dyrk1A. The compound adopts an approximately co-planar conformation with a good shape complementarity to the binding cleft. (B) Top view of the ATP pocket from the N-terminus. Compound **29** is anchored between Lys188 and Leu241 via two hydrogen bonds. Two CH–π interactions are formed with Val306 and a Lys188 methylene, respectively, and two with Phe238. (C) Binding mode of harmine within the ATP pocket. Harmine targets Lys188 and Leu241 via hydrogen bonds as well but establishes only one CH–π interaction in the crystal structure (with Val306). Blue: nitrogen, green: sulfur, red: oxygen, light grey: receptor carbon atoms, white: carbon atoms of **29**, orange: carbon atoms of harmine, hydrogen is omitted for clarity. Receptor surface is cut in B and C.

To get a better insight in the binding mechanism of our compound class, we performed a multi-step *in silico* study employing local docking and molecular dynamics simulations with our lead compound **29**. The cocrystal structure of harmine and Dyrk1A was used as a template (PDB accession Code: 3ANR). The results were compared with the binding mode of harmine within the ATP pocket. As depicted in Figure 9, compound **29** is anchored between the conserved Lys188 and the hinge region residue Leu241 via two hydrogen bonds. In addition, we found two CH–π interactions involving Lys188 and Val306 and two edge-to-face CH–π interactions with the Phe238 benzene ring. Interestingly, **29** only takes advantage of the adenine- and the phosphate-binding regions, offering a high potential to expand the inhibitor to target additional areas within the ATP pocket. These further ligand modifications could enhance the potency but also the selectivity within the Dyrk family and against Clk1 if needed. The predicted binding pose for **29** also provided another potential explanation why the pyridine analogue of **29**, compound **4**, was considerably less active: for steric reasons, the larger pyridine ring is expected to prevent a similarly deep docking in the ATP pocket, thus impairing the CH–π interactions with the Phe238 benzene ring.

The cocrystalized harmine exploits the same hydrogen bonds to Dyrk1A. However, we could detect only one CH–π interaction between Val306 and harmine (Figure 9C). These less extensive interactions with the aliphatic side chains and Phe238 are probably compensated by the energetically more favorable angles of the hydrogen bonds established by harmine. A second reason could be the higher hydrogen bond acceptor strength of 2-methylpyridine (pK_BHX_ : 2.03) compared to 1,3-thiazole (pK_BHX_ : 1.37), compensating for the fewer CH–π interactions [86].

### Selectivity profile

During lead optimization, we routinely determined the inhibitory activity towards three Dyrk isoforms and against CK2α as a primary counter screen. The latter kinase was not appreciably inhibited by any of the compounds. Further frequently reported off-targets for Dyrk inhibitors comprise Clks, PIM1, HIPK2 and PKD2 [56], [81], [82], [97]. Therefore, we tested the selectivity of our five most potent compounds especially against these kinases and in addition against further kinases representing each subfamily of the human kinome (Tables S3 and S4, and Figures S3 and S4 in File S1). Table S4 contains an extended kinase list to assess the selectivity of the most potent compounds **29** and **48**. As can be seen from Tables S3 and S4, no other kinase was inhibited to the same degree as the Dyrk kinases, with the exception of Clk1/4. This coupled inhibitory activity was consistent with former reports: all Dyrk1A and Dyrk1B inhibitors as published to date, and for which selectivity data were provided, also displayed Clk1 inhibition with similar potency, while other kinases were affected at variable degrees [17]–[20], [84], [98]. This was also true for harmine, which showed an IC_50_ for Clk1 of 0.2 µM (Table 9). Hence, our lead compounds were still among the most selective Dyrk inhibitors. In the case of compound **29**, the MAP kinase MNK1 was also markedly inhibited (83% at 5 µM). Interestingly, this inhibitory activity was rather abolished in compound **48**, which differs from **29** only by the relative position of the thiophene sulfur. In the light of this rather unexpected finding, it might be worth investigating the role of the thiophene sulfur in the generation of inhibitory activity against MNK1, and also to screen our library to identify further potential inhibitors of MNK1 with reduced activity against Dyrks and Clks. MNK1 was validated as a potential target in glioblastoma by Brian Hemmings' group [99]. Since Dyrk1A was also proposed as a new target in EGFR-dependent glioblastoma [38], compound **29** might even be considered a new lead for the development of dual Dyrk1A/MNK1 inhibitors to defeat this aggressive subset of brain tumors. The next most strongly inhibited kinase in our panel was PIM1. The IC_50_ values of **30**, **33** and **48** for PIM1 were all about 5 µM, which was, e.g. in the case of compound **48**, 50 times higher than the IC_50_ for Dyrk1B inhibition.

**Table 9:** Potency of the dual Dyrk/Clk inhibitors 29, 30, 33, 41 and 48 against the target kinases.

Kinases		29	30	33	41	48	Harmine
Dyrk1A	IC_50_ [nM]	130	300	810	880	100	100
	% inhibition 5 µM	90%	99%	84%	85%	97%	98%
Dyrk1B	IC_50_ [nM]	100	410	1000	1800	70	228
	% inhibition 5 µM	100%	100%	83%	70%	100%	98%
Dyrk2	IC_50_ [nM]	430	190	1300	3400	40	3000
	% inhibition 5 µM	93%	100%	77%	57%	100%	68%
Clk1	IC_50_ [nM]	130	160	420	610	110	220
	% inhibition 5 µM	96%	92%	88%	86%	94%	97%

a Given are mean values of at least two independent experiments, S.D.<10%.

Altogether, the new inhibitors displayed a good selectivity for the Dyrk family of kinases, qualifying them as promising lead compounds.

### Relative potencies within the Dyrk/Clk target family

Among the most potent compounds **29** and **48**, **48** was slightly more active against Dyrk1B than against Clk1, while **29** was equally potent towards Dyrk1A, 1B and Clk1, but exhibited some selectivity against Dyrk2 (Table 9). However, in the case of **33**, Clk1 was the main target (though the potency was only moderate). A further extension of the 5-thiophenyl oxazole series, to which **33** belongs, might thus yield selective Clk1 inhibitors. In the larger selectivity screening, Clk4 was identified as an additional target within the Clk family (see Table S4 in File S1 and below), whereas Clk2 and −3 were only slightly affected – if at all. Based on the high degree of identity between Clk1 and −4 (but not between Clk1 and Clk2/3), similar potencies of our compounds can be expected towards Clk1 and Clk4.

## Conclusions

The novel kinase inhibitor class of 2,4-bisheterocyclic substituted thiophenes possesses a flexible scaffold, which allowed the independent optimization of both molecule ends using a large set of commercially available heterocyclic moieties, consisting of five- and six-membered rings and annelated ring systems. Automated parallel synthesis of several focused libraries of compounds accelerated the optimization of the potency and selectivity while increasing the ligand efficiency, thus resulting in lead-like molecules [100]. Our two most potent inhibitors, **29** and **48**, exhibited an activity towards Dyrk1A similar to that of the first-in-class inhibitor harmine (Table 9), however, at a somewhat reduced cytotoxic potential and with higher metabolic stability.

Within the tested panel of kinases, the inhibitors displayed a good selectivity for the ATP- binding pockets of the Dyrk and Clk1/4 family. This was probably achieved by a combination of the following strategies: (i) Establishment of only one hydrogen bond each with the hinge region and the conserved lysine, respectively; thus, even small shifts in the optimum spatial arrangements of the donor moieties in the protein will result in a large drop of the binding affinity. (ii) Generation of the optimum shape and electrostatic complementarity by identifying the appropriate combination of azaheterocycles in our parallel synthesis campaign. Further fine-tuning of potency and selectivity was achieved by a simple change of the relative position of the thiophene sulfur. Both isomeric compounds **29** and **48** might serve as potential leads for the development of new anti-cancer therapeutics due to their inhibitory activity against Dyrk1A and −1B, which we also demonstrated in intact cells; **29** might offer an additional advantage in the case of glioblastoma, due to the simultaneous inhibition of Dyrk1A and Mnk1, which were both validated as new promising targets for this tumor entity [38], [99]. Furthermore, compound **24** was selective for Dyrk2 (IC_50_ = 0.63 µM) among the three Dyrk family members tested. Thus, we anticipate that with our focused library approach, it might also be possible to generate inhibitors of the Dyrk3 and 4 isoforms as well, for which no chemical inhibitors have been reported yet, and no crystal structures are available. So far it is not clear whether selectivity against Clk1 can be achieved at all for ATP-competitive Dyrk inhibitors. However, the dual Dyrk1A/Clk1 inhibitory activity might even provide higher efficacy for the treatment of tauopathies, because both kinases are modifying the pre-mRNA splicing to generate tau species with enhanced pathogenic potential.

## Supporting Information

File S1 **Combined Supporting Information File S1 (containing Figures S1, S2 and S3 and Tables S1, S2, S3 and S4).** **Figure S1.** Overlay of compound **1** (grey), compound **4** (orange) and harmine (green). The hydrogen bond acceptor atoms of **4** occupy similar positions compared to harmine, whereas the hydroxyl groups of compound **1** address different spatial positions. **Figure S2.** Inhibition of Dyrk1A by compound **4** is competitive with respect to the co-factor ATP. The Michaelis-Menten curves in the absence (A) vs. the presence of inhibitor **4** (B) indicate an ATP-competititive binding mechanism. The V_max_ of both kinase reactions was 0.051 µmol/l*min. For the kinetic experiment, kinase assays were performed with 500 nM of compound **4** or DMSO as described in the Experimental Section using ten different ATP concentrations as follows (µM; cold ATP+γ-^32^ATP): 1+0.003, 5+0.017, 10+0.033, 20+0.066, 50+0.165, 75+0.248, 150+0.5, 250+0.825, 500+1.65. The kinase reactions were performed at 30°C for 2.5 min and terminated by spotting 5 µl of the reaction mixture onto P81 phosphocellulose paper, which was further treated as decribed. K_m_ and V_max_ values were calculated by fitting the data with Origin Pro 8.6 (OriginLabs). Error bars denote the standard deviation of mean. **Figure S3.** Overlay of the quinoline derivative **20** and the isoquinoline **21**. a) represents the identical part of both compounds; b) additional hydrophobic part of **20** compared with **4**; c) additional hydrophobic part of **21**. Assuming that the same set of hydrogen bonds is formed with the nitrogen atoms, the overlay suggests that parts b) and c) are accommodated by different hydrophobic areas within the ATP-binding pocket. **Figure S4.** The selectivity of compound **29** is illustrated in a kinome tree dendrogram. The kinases tested are highlighted in green, orange or red circles corresponding to their inhibition at 5 µM (green: 0–40% inhibition, orange: 40–80% inhibition, red: >80% inhibition). **Figure S5.** The selectivity of compound **48** is illustrated in a kinome tree dendrogram. The kinases tested are highlighted in green, orange or red circles corresponding to their inhibition at 5 µM (green: 0–40% inhibition, orange: 40–80% inhibition, red: >80% inhibition). **Table S1.** Calculated Ligand efficiencies (LE) of the most potent compounds against Dyrk1A and Dyrk1B. **Table S2.** Calculated molecular properties of the most potent compounds compared to harmine. **Table S3.** Selectivity panel of compounds **29**, **30**, **33**, **41** and **48** against selected kinases frequently reported to be affected by Dyrk inhibitors*^a^*. **Table S4.** Extended selectivity panel for compound **29** and **30** *^a^*.
(ZIP)

File S2 **Combined Supporting Information File S2 (containing additional experimental procedures and analytical data).** **1**) Synthetic procedures and analytical data of compounds **2**, **3**, **6**, **7**, **9–14**, **16**, **18**, **21–24**, **26–28**, **31**, **32**, **34–40**, **42–47**. **2**) Construction of the pET45b-Dyrk1A-cd expression plasmid.
(ZIP)
