# Supplementary figures and images for: Design and Synthesis of a Library of Lead-Like 2,4-Bisheterocyclic Substituted Thiophenes as Selective Dyrk/Clk Inhibitors
Source: PLoS One. 2014 Mar 27;9(3):e87851. doi: 10.1371/journal.pone.0087851 (PMC3968014; doi:10.1371/journal.pone.0087851)

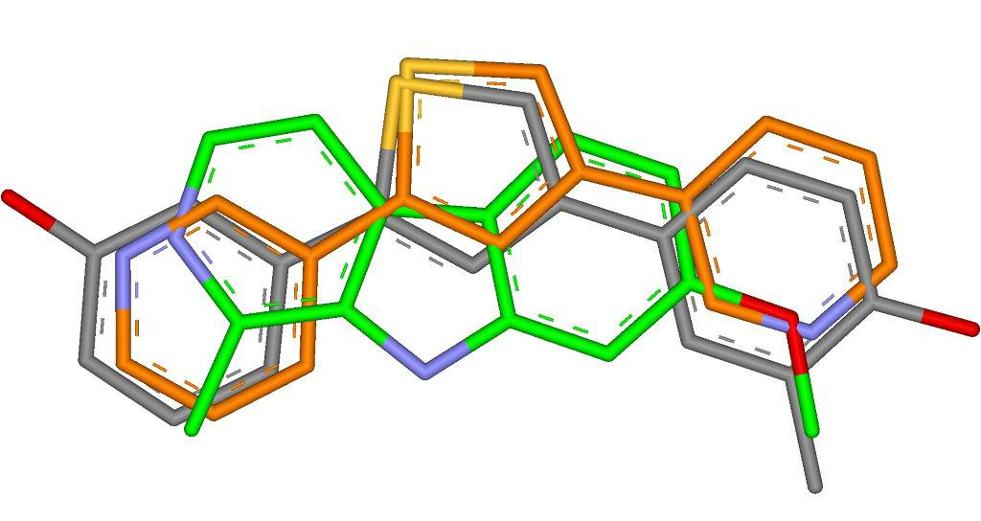

Supplement: File S1 — Combined Supporting Information File S1 (containing Figures S1, S2 and S3 and Tables S1, S2, S3 and S4). Figure S1. Overlay of compound 1 (grey), compound 4 (orange) and harmine (green). The hydrogen bond acceptor atoms of 4 occupy similar positions compared to harmine, whereas the hydroxyl groups of compound 1 address different spatial positions. Figure S2. Inhibition of Dyrk1A by compound 4 is competitive with respect to the co-factor ATP. The Michaelis-Menten curves in the absence (A) vs. the presence of inhibitor 4 (B) indicate an ATP-competititive binding mechanism. The Vmax of both kinase reactions was 0.051 µmol/l*min. For the kinetic experiment, kinase assays were performed with 500 nM of compound 4 or DMSO as described in the Experimental Section using ten different ATP concentrations as follows (µM; cold ATP+γ-32ATP): 1+0.003, 5+0.017, 10+0.033, 20+0.066, 50+0.165, 75+0.248, 150+0.5, 250+0.825, 500+1.65. The kinase reactions were performed at 30°C for 2.5 min and terminated by spotting 5 µl of the reaction mixture onto P81 phosphocellulose paper, which was further treated as decribed. Km and Vmax values were calculated by fitting the data with Origin Pro 8.6 (OriginLabs). Error bars denote the standard deviation of mean. Figure S3. Overlay of the quinoline derivative 20 and the isoquinoline 21. a) represents the identical part of both compounds; b) additional hydrophobic part of 20 compared with 4; c) additional hydrophobic part of 21. Assuming that the same set of hydrogen bonds is formed with the nitrogen atoms, the overlay suggests that parts b) and c) are accommodated by different hydrophobic areas within the ATP-binding pocket. Figure S4. The selectivity of compound 29 is illustrated in a kinome tree dendrogram. The kinases tested are highlighted in green, orange or red circles corresponding to their inhibition at 5 µM (green: 0–40% inhibition, orange: 40–80% inhibition, red: >80% inhibition). Figure S5. The selectivity of compound 48 is illustrated i [file pone.0087851.s001.zip › Figure_S1.tif]

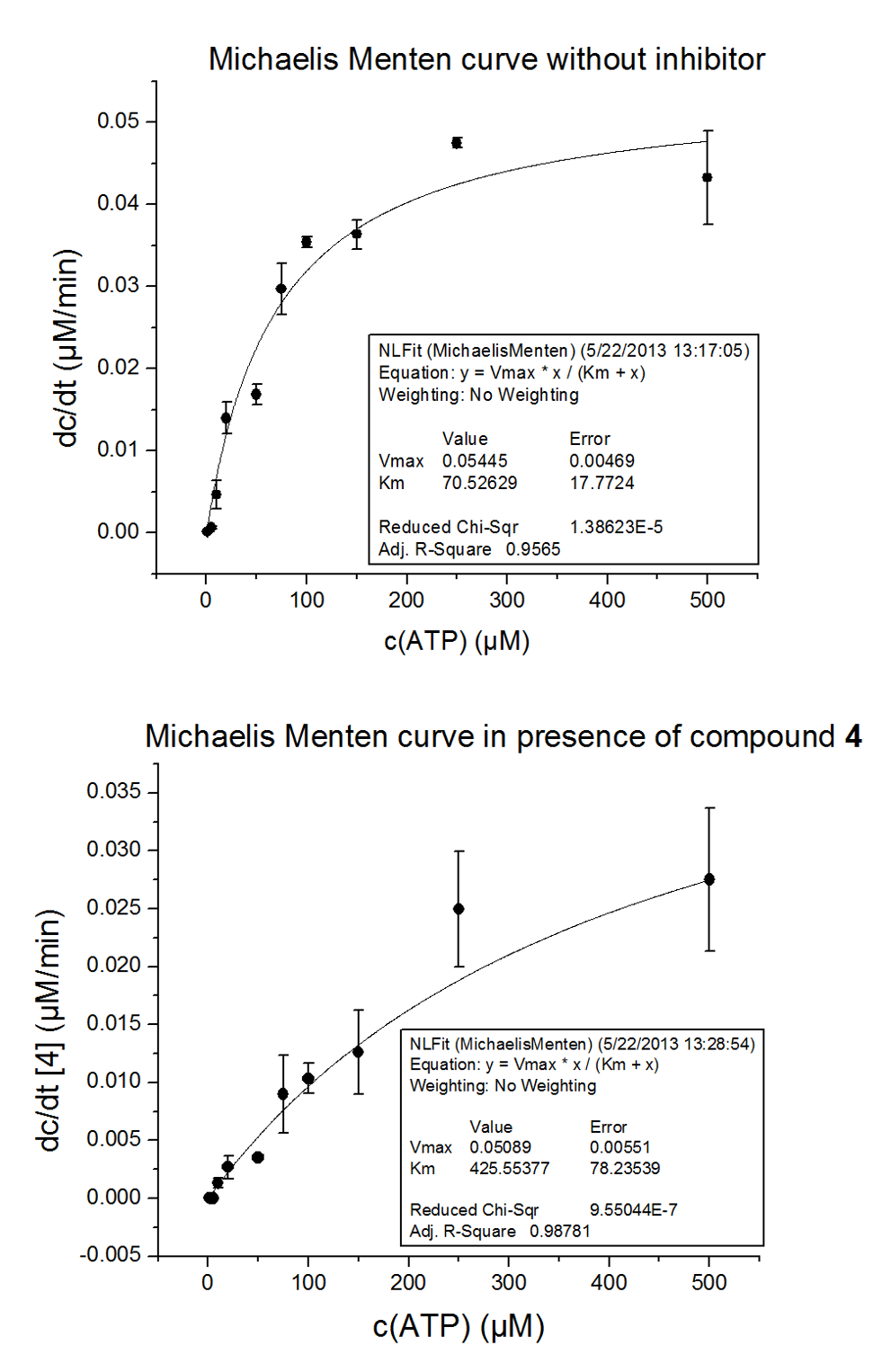

Supplement: File S1 — Combined Supporting Information File S1 (containing Figures S1, S2 and S3 and Tables S1, S2, S3 and S4). Figure S1. Overlay of compound 1 (grey), compound 4 (orange) and harmine (green). The hydrogen bond acceptor atoms of 4 occupy similar positions compared to harmine, whereas the hydroxyl groups of compound 1 address different spatial positions. Figure S2. Inhibition of Dyrk1A by compound 4 is competitive with respect to the co-factor ATP. The Michaelis-Menten curves in the absence (A) vs. the presence of inhibitor 4 (B) indicate an ATP-competititive binding mechanism. The Vmax of both kinase reactions was 0.051 µmol/l*min. For the kinetic experiment, kinase assays were performed with 500 nM of compound 4 or DMSO as described in the Experimental Section using ten different ATP concentrations as follows (µM; cold ATP+γ-32ATP): 1+0.003, 5+0.017, 10+0.033, 20+0.066, 50+0.165, 75+0.248, 150+0.5, 250+0.825, 500+1.65. The kinase reactions were performed at 30°C for 2.5 min and terminated by spotting 5 µl of the reaction mixture onto P81 phosphocellulose paper, which was further treated as decribed. Km and Vmax values were calculated by fitting the data with Origin Pro 8.6 (OriginLabs). Error bars denote the standard deviation of mean. Figure S3. Overlay of the quinoline derivative 20 and the isoquinoline 21. a) represents the identical part of both compounds; b) additional hydrophobic part of 20 compared with 4; c) additional hydrophobic part of 21. Assuming that the same set of hydrogen bonds is formed with the nitrogen atoms, the overlay suggests that parts b) and c) are accommodated by different hydrophobic areas within the ATP-binding pocket. Figure S4. The selectivity of compound 29 is illustrated in a kinome tree dendrogram. The kinases tested are highlighted in green, orange or red circles corresponding to their inhibition at 5 µM (green: 0–40% inhibition, orange: 40–80% inhibition, red: >80% inhibition). Figure S5. The selectivity of compound 48 is illustrated i [file pone.0087851.s001.zip › Figure_S2.tif]

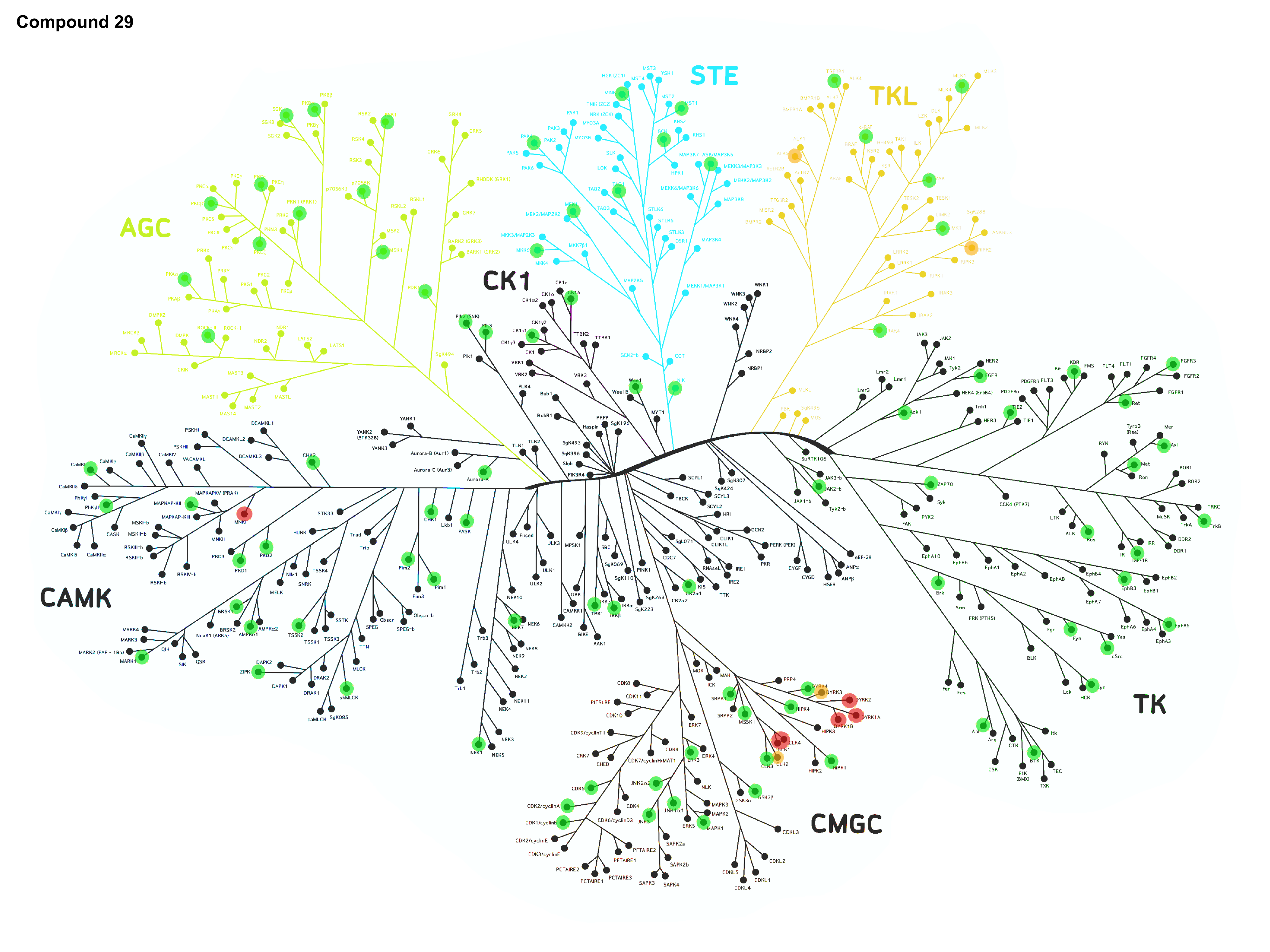

Supplement: File S1 — Combined Supporting Information File S1 (containing Figures S1, S2 and S3 and Tables S1, S2, S3 and S4). Figure S1. Overlay of compound 1 (grey), compound 4 (orange) and harmine (green). The hydrogen bond acceptor atoms of 4 occupy similar positions compared to harmine, whereas the hydroxyl groups of compound 1 address different spatial positions. Figure S2. Inhibition of Dyrk1A by compound 4 is competitive with respect to the co-factor ATP. The Michaelis-Menten curves in the absence (A) vs. the presence of inhibitor 4 (B) indicate an ATP-competititive binding mechanism. The Vmax of both kinase reactions was 0.051 µmol/l*min. For the kinetic experiment, kinase assays were performed with 500 nM of compound 4 or DMSO as described in the Experimental Section using ten different ATP concentrations as follows (µM; cold ATP+γ-32ATP): 1+0.003, 5+0.017, 10+0.033, 20+0.066, 50+0.165, 75+0.248, 150+0.5, 250+0.825, 500+1.65. The kinase reactions were performed at 30°C for 2.5 min and terminated by spotting 5 µl of the reaction mixture onto P81 phosphocellulose paper, which was further treated as decribed. Km and Vmax values were calculated by fitting the data with Origin Pro 8.6 (OriginLabs). Error bars denote the standard deviation of mean. Figure S3. Overlay of the quinoline derivative 20 and the isoquinoline 21. a) represents the identical part of both compounds; b) additional hydrophobic part of 20 compared with 4; c) additional hydrophobic part of 21. Assuming that the same set of hydrogen bonds is formed with the nitrogen atoms, the overlay suggests that parts b) and c) are accommodated by different hydrophobic areas within the ATP-binding pocket. Figure S4. The selectivity of compound 29 is illustrated in a kinome tree dendrogram. The kinases tested are highlighted in green, orange or red circles corresponding to their inhibition at 5 µM (green: 0–40% inhibition, orange: 40–80% inhibition, red: >80% inhibition). Figure S5. The selectivity of compound 48 is illustrated i [file pone.0087851.s001.zip › Figure_S4.tif]

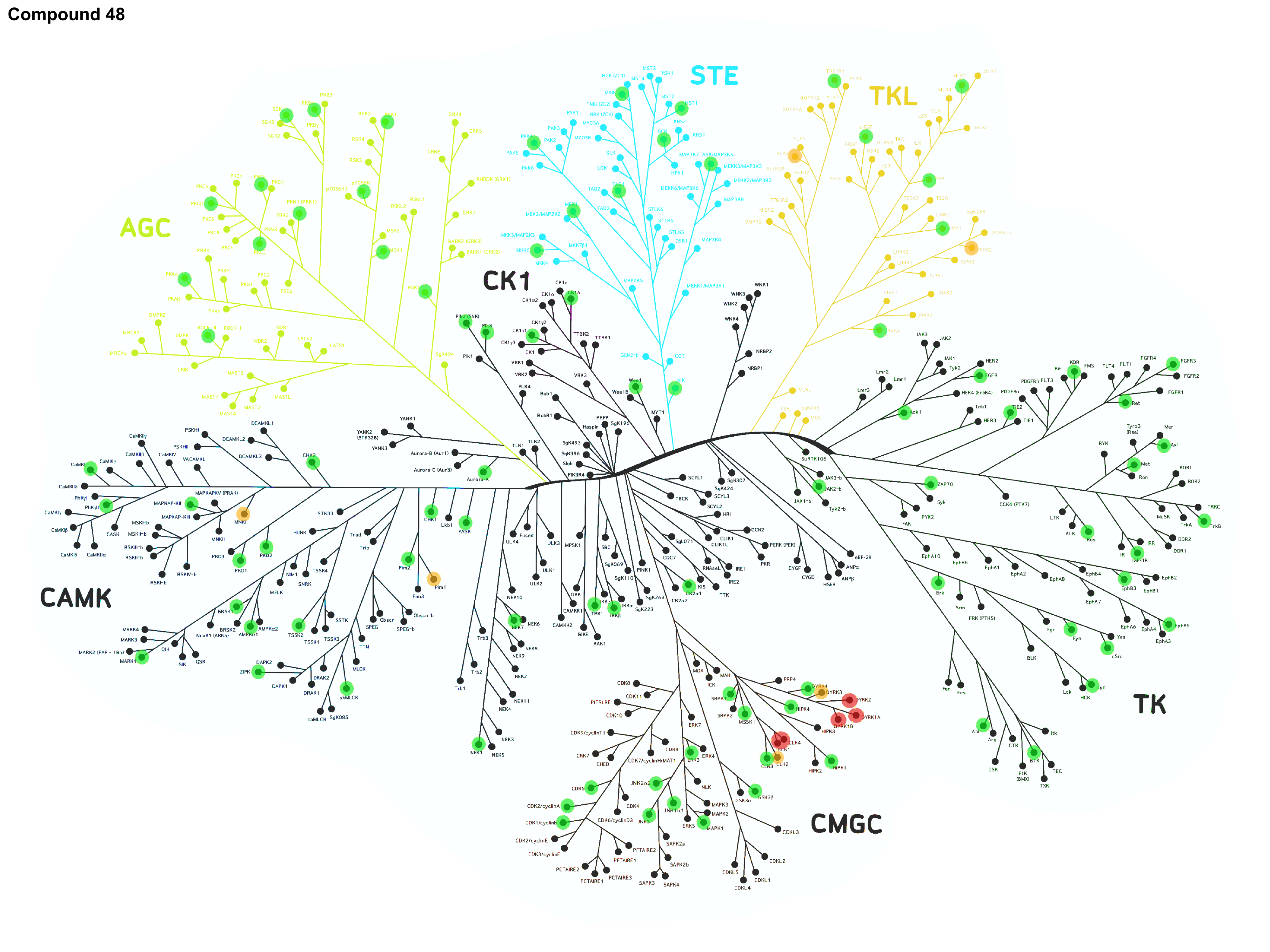

Supplement: File S1 — Combined Supporting Information File S1 (containing Figures S1, S2 and S3 and Tables S1, S2, S3 and S4). Figure S1. Overlay of compound 1 (grey), compound 4 (orange) and harmine (green). The hydrogen bond acceptor atoms of 4 occupy similar positions compared to harmine, whereas the hydroxyl groups of compound 1 address different spatial positions. Figure S2. Inhibition of Dyrk1A by compound 4 is competitive with respect to the co-factor ATP. The Michaelis-Menten curves in the absence (A) vs. the presence of inhibitor 4 (B) indicate an ATP-competititive binding mechanism. The Vmax of both kinase reactions was 0.051 µmol/l*min. For the kinetic experiment, kinase assays were performed with 500 nM of compound 4 or DMSO as described in the Experimental Section using ten different ATP concentrations as follows (µM; cold ATP+γ-32ATP): 1+0.003, 5+0.017, 10+0.033, 20+0.066, 50+0.165, 75+0.248, 150+0.5, 250+0.825, 500+1.65. The kinase reactions were performed at 30°C for 2.5 min and terminated by spotting 5 µl of the reaction mixture onto P81 phosphocellulose paper, which was further treated as decribed. Km and Vmax values were calculated by fitting the data with Origin Pro 8.6 (OriginLabs). Error bars denote the standard deviation of mean. Figure S3. Overlay of the quinoline derivative 20 and the isoquinoline 21. a) represents the identical part of both compounds; b) additional hydrophobic part of 20 compared with 4; c) additional hydrophobic part of 21. Assuming that the same set of hydrogen bonds is formed with the nitrogen atoms, the overlay suggests that parts b) and c) are accommodated by different hydrophobic areas within the ATP-binding pocket. Figure S4. The selectivity of compound 29 is illustrated in a kinome tree dendrogram. The kinases tested are highlighted in green, orange or red circles corresponding to their inhibition at 5 µM (green: 0–40% inhibition, orange: 40–80% inhibition, red: >80% inhibition). Figure S5. The selectivity of compound 48 is illustrated i [file pone.0087851.s001.zip › Figure_S5.tif]
