# Supplementary material for: Design and Synthesis of a Library of Lead-Like 2,4-Bisheterocyclic Substituted Thiophenes as Selective Dyrk/Clk Inhibitors
Source: PLoS One. 2014 Mar 27;9(3):e87851. doi: 10.1371/journal.pone.0087851 (PMC3968014; doi:10.1371/journal.pone.0087851)
Supplement: File S2 — Combined Supporting Information File S2 (containing additional experimental procedures and analytical data). 1) Synthetic procedures and analytical data of compounds 2, 3, 6, 7, 9–14, 16, 18, 21–24, 26–28, 31, 32, 34–40, 42–47. 2) Construction of the pET45b-Dyrk1A-cd expression plasmid. (ZIP) [file pone.0087851.s002.zip › File_S2.docx]

Design and Synthesis of a library of lead-like 2,4-bisheterocyclic substituted thiophenes as selective Dyrk/Clk inhibitors.

*Christian Schmitt^1^, Dagmar Kail^2^, Marica Mariano^1^, Martin Empting^3^, Nadja Weber^1^, Tamara Paul^1^, Rolf W. Hartmann ^1,3^, Matthias Engel^1^*

AUTHOR ADDRESS

^1^Pharmaceutical and Medicinal Chemistry, Saarland University, Campus C2.3, D-66123 Saarbrücken, Germany

^2^PharmBioTec GmbH, Uni-Campus Nord 2, D-66123 Saarbrücken, Germany

^3^Department of Drug Design and Optimization, Helmholtz-Institut für Pharmazeutische Forschung Saarland, Campus C2.3, D-66123 Saarbrücken, Germany

**Supporting Information (S2)**

**Contents:**

1. Synthetic procedures and analytical data of compounds **2**, **3**, **6, 7, 9-14, 16, 18, 21-24, 26-28, 31, 32, 34-40, 42-47**………………………………………………….. *Page S2*
2. Construction of the pET45b-Dyrk1A-cd expression plasmid……………….. *Page S17*
3. Literature………………………………………………………………………P*age S19*

**1) Synthetic procedures and analytical data of compounds 2, 3, 6, 7, 9-14, 16, 18, 21-24, 26-28, 31, 32, 34-40, 42-47**

*Procedure for the synthesis of 4-bromo-2-(3-methoxyphenyl)thiophene* **2a**: 5.5 ml of n-buthyllithium (2.5 M in hexane, 13.6 mmol) was added slowly to a solution of 2g (10.7 mmol) of 3-bromoanisole in 40 ml THF at -78°C under nitrogen. The mixture was stirred for 60 min and 3.56 g (15.5 mmol, 4.17 ml) of tributyl borate was added dropwise. The temperature should not exceed -70°C. The mixture was stirred at -78°C for another 90 min before it was allowed to warm to room temperature. Then, 2.3 g (21.4 mmol) of sodium carbonate dissolved in 10 ml of water, 0.49 g (0.43 mmol) of tetrakis(triphenylphosphine)palladium(0) and 2.37 g (10 mmol) of 2,4-dibromothiophene were added and the mixture was heated to reflux overnight. The reaction was stopped when no starting material could be detected. The crude mixture was dissolved in 40 ml of water extracted with diethyl ether (4x). The combined organic layer was subsequently washed with water and brine, dried over magnesium sulfate and the solvent was removed under reduced pressure. The crude product was purified by flash column chromatography eluting with ethyl acetate/hexane 1:30 to give 1.79 g (6.7 mmol), 63 % of **2a**. ^1^H NMR (500 MHz, DMSO-*d*_6_) δ ppm 3.76 - 3.86 (m, 3 H) 6.93 (ddd, *J*=8.20, 2.36, 0.79 Hz, 1 H) 7.17 - 7.27 (m, 2 H) 7.30 - 7.39 (m, 1 H) 7.60 (d, *J*=1.26 Hz, 1 H) 7.67 (d, *J*=1.26 Hz, 1 H); ^13^C NMR (126 MHz, DMSO-*d*_6_) δ ppm 55.26, 109.79, 110.63, 114.19, 117.68, 123.15, 126.14, 130.31, 133.80, 144.58, 159.79; Purity(FID): 95 %, MS(EI), m/z [M]^+^: 267.88, calc. 267.96.

*Procedure for the synthesis of 3-methoxy-5-methylphenyl trifluoromethanesulfonate*[1] **2b**: 1 g (7.24 mmol) of 3-methoxy-5-methylphenol was dissolved in dichloromethane under nitrogen atmosphere and cooled to 0°C. 2.45 g (8.7 mmol) of trifluoromethanesulfonic anhydride were added dropwise and the mixture was allowed to warm to room temperature. The reaction progress was monitored by TLC. After completion, the crude mixture was dissolved in diethyl ether and poured into 4 % HCl. The organic phase was washed subsequently with brine and saturated sodium hydrogen carbonate. The organic layer was dried over magnesium sulfate, reduced under reduced pressure. The resulting product was sufficiently pure (purity > 95 %) and used without further purification, yield: 1.67 g (6.2 mmol) 85 %. ^1^H NMR (500 MHz, DMSO-*d*_6_) δ ppm 2.50 (d, *J*=0.63 Hz, 3 H) 3.95 (s, 3 H) 7.00 - 7.04 (m, 2 H) 7.06 - 7.10 (m, 1 H); ^13^C NMR (126 MHz, DMSO-*d*_6_) δ ppm 20.91, 55.75, 104.64, 113.51, 115.13, 141.60, 149.62, 160.30, 1 C not detected; Purity(FID): 95 %, MS(EI), m/z [M]^+^: 269.87, calc. 270.02.

*Procedure for the synthesis of 2-(3-methoxy-5-methylphenyl)-4,4,5,5-tetramethyl-1,3,2-dioxaborolane* **2c**: 0.2 g (0.25 mmol) of [1,1′-bis(diphenylphosphino)ferrocene] dichloropalladium(II) were suspended in dry dioxane under nitrogen. 1.67 g (6.2 mmol) of **2b**, 1.88 g (18.6 mmol) of triethylamine and 2.36 g (9.3 mmol) of bis(pinacolato)diboron were added successively. The mixture was heated to reflux until the reaction was completed. Then 10 ml of water was added and the crude product was dissolved in 50 ml of dichloromethane. The organic layer was washed with water and brine, dried over magnesium sulfate and the solvent was removed under reduced pressure. The crude product was purified by flash column chromatography eluting with ethyl acetate/hexane 1:10 to yield 0.81 g (3.27 mmol), 53 % of **2c**. ^1^H NMR (500 MHz, DMSO-*d*_6_) δ ppm 1.50 - 1.52 (m, 15 H) 3.96 (s, 3 H) 7.08 - 7.10 (m, 1 H) 7.18 (d, *J*=2.84 Hz, 1 H) 7.31 (d, *J*=0.63 Hz, 1 H); ^13^C NMR (126 MHz, DMSO-*d*_6_) δ ppm 24.28, 54.81, 83.57, 115.57, 118.19, 127.57, 134.35, 138.54, 158.80; Purity(FID): 98 %, MS(EI), m/z [M]^+^: 248.03, calc. 248.16.

*Procedure for the synthesis of 4-(3-methoxy-5-methylphenyl)-2-(3-methoxyphenyl)thiophene* **2d**: 0.15 g (0.56 mmol) of **2a** was dissolved in 10 ml of dioxane/water 5:1 under nitrogen. To this solution 0.14 g (0.56 mmol) of **2c**, 0.12 g (1.12 mmol) of sodium carbonate and 0.032 g (0.028 mmol) of tetrakis(triphenylphosphine)palladium(0) were subsequently added and the mixture was heated to reflux. The reaction was monitored by TLC. The crude mixture was poured into water and extracted with diethyl ether (4x). The combined organic layer was washed with brine, dried over magnesium sulfate and the solvent was removed in vacuo. The crude product was purified by flash column chromatography eluting with ethyl acetate/hexane 1:25 to yield 0.164 g (0.53 mmol), 93 % of **2d**; ^1^H NMR (500 MHz, Methanol-*d*_4_) δ ppm 2.25 (s, 3 H) 3.72 (s, 3 H) 3.74 (s, 3 H) 6.57 - 6.60 (m, 1 H) 6.75 - 6.78 (m, 1 H) 6.89 - 6.92 (m, 1 H) 6.97 - 7.01 (m, 1 H) 7.08 - 7.11 (m, 1 H) 7.12 - 7.16 (m, 1 H) 7.17 - 7.21 (m, 1 H) 7.40 (d, *J*=1.58 Hz, 1 H) 7.57 (d, *J*=1.58 Hz, 1 H); ^13^C NMR (126 MHz, Methanol-*d*_4_) δ ppm 21.67, 55.72, 55.80, 110.06, 112.25, 114.22, 114.55, 119.23, 120.58, 120.83, 123.60, 131.06, 137.04, 138.23, 140.99, 144.48, 161.61, 161.66; Purity(FID): 98 %, MS(EI), m/z [M]^+^: 309.94, calc. 310.10.

*Procedure for the synthesis of 3-(5-(3-hydroxyphenyl)thiophen-3-yl)-5-methylphenol* **2**: A solution of 0.15 g (0.48 mmol) of **2d** in dry dichloromethane was cooled to -78°C and 2.9 mmol BBr_3_ was carefully added (2.9 ml of a 1M solution in dichloromethane). The mixture was allowed to warm to room temperature. The reaction was stirred for 4 hours and the reaction was stopped by addition of 10 ml of water. The mixture was extracted with ethyl acetate and the combined organic layer was dried over magnesium sulfate. The solvent was removed under reduced pressure and purified by flash column chromatography to give 74 mg of **2** (54 %). ^1^H NMR (300 MHz, Acetone) δ ppm 2.31 (s, 3 H) 6.65 (s, 1 H) 6.82 (dt, *J*=7.26, 2.14 Hz, 1 H) 7.00 - 7.02 (m, 1 H) 7.08 (dt, *J*=1.68, 1.02 Hz, 1 H) 7.19 - 7.29 (m, 3 H) 7.59 (d, *J*=1.49 Hz, 1 H) 7.75 (d, *J*=1.49 Hz, 1 H); ^13^C NMR (75 MHz, Acetone) δ ppm 20.63, 110.19, 110.28, 114.68, 115.00, 116.92, 118.42, 119.54, 122.46, 130.12, 135.63, 136.85, 139.63, 143.19, 144.51, 157.68, 157.92; Purity(FID): 98 %, MS(EI), m/z [M]^+^: 282.01, calc. 282.07.

*Procedure for the synthesis of 4-bromo-2-(4-methoxy-3-methylphenyl)thiophene* **3a**: A solution of 0.83 g (4.14 mmol) of 4-bromo-2-methylanisole in 15 ml dry, nitrogen flushed THF was cooled to -78°C. A 2.5 M solution of n-buthyllithium (1.82 ml) was carefully added and the mixture was stirred for 60 min at -78°C. Then, 0.85 g (5 mmol) of tributyl borate was slowly added and the mixture was stirred for additional 90 min at -78°C. Afterwards the mixture was allowed to warm to room temperature and 1.1 g (10.35 mmol) of sodium carbonate in 2 ml of water, 1 g (4.14 mmol) of 2,4-dibromothiophene and 0.19 g (0.16 mmol) of tetrakis(triphenylphosphine)palladium(0) was added. The mixture was heated to reflux and the reaction was monitored by TLC. The reaction was stopped when all starting materials were consumed and the crude reaction mixture was poured into water. The crude product was extracted with diethyl ether (4x) and the combined organic layer was washed with water and brine. The solvent was dried over magnesium sulfate and removed in vacuo. The crude product was purified by flash column chromatography eluting with ethyl acetate/hexane 1:30 to give 0.82 g (2.9 mmol), 70 % of **3a** as a pale yellow solid. ^1^H NMR (500 MHz, Methanol-*d*_4_) δ ppm 2.11 (s, 3 H) 3.74 (s, 3 H) 6.80 (d, *J*=8.51 Hz, 1 H) 7.05 (d, *J*=1.58 Hz, 1 H) 7.13 (d, *J*=1.58 Hz, 1 H) 7.25 - 7.30 (m, 2 H); ^13^C NMR (126 MHz, Methanol-*d*_4_) δ ppm 16.30, 55.92, 111.23, 111.41, 122.00, 125.41, 125.44, 128.35, 128.87, 132.10, 132.48, 159.51; Purity(FID): 97 %, MS(EI), m/z [M]^+^: 281.89, calc. 281.97.

*Procedure for the synthesis of 2-(4-methoxy-3-methylphenyl)-4-(3-methoxyphenyl)thiophene* **3b**: A solution of 0.13 g (0.7 mmol) of 3-bromoanisole in 10 ml of dry THF was cooled to -78°C and 0.3 ml of n-buthyllithium (2.5 M in hexane) was added dropwise. After 60 min, 0.19 g (0.85 mmol) of tributyl borate was added and the mixture was stirred for additional 90 min at -78°C. Then, the mixture was allowed to warm to room temperature and 0.2 g (0.7 mmol) of **3a**, 0.19 g (1.8 mmol) of sodium carbonate in 2 ml of water and 0.023 mg (0.028 mmol) of [1,1′-bis(diphenylphosphino)ferrocene] dichloropalladium(II) were added. The mixture was heated to reflux and the reaction was monitored by TLC. The reaction was stopped when no starting materials were detected and the reaction mixture was poured into water. The crude product was extracted with ethyl acetate, washed with water and brine and dried over magnesium sulfate. The solvent was evaporated under reduced pressure and the resulting yellow oil was purified by flash column chromatography to yield 0.17 g (0.55 mmol) of **3b** as white solid. 1H-NMR (Methanol-d_4_) δ ppm: 7,43 (d, J = 1,48 Hz, 1H), 7,35-7,33 (m, 3H), 7,19 (t, J = 7,85 Hz, 1H), 7,14-7,12 (m, 1H), 7,10-7,09 (m, 1H), 6,79 (d, J = 8,14 Hz, 1H), 6,75-6,73 (ddd, J1 = 8,08 Hz, J2 = 2,55 Hz, J3 = 1,03 Hz, 1H), 3,73 (s, 6H), 2,12 (s, 3H); 13C-NMR (Methanol-d_4_) δ ppm: 161,59, 159,06, 146,42, 144,18, 138,69, 130,82, 128,99, 128,1, 128,06, 125,44, 122,04, 119,72, 113,61, 112,86, 111,35, 55,89, 55,74, 16,35; Purity(FID): 98 %, MS(EI), m/z [M]^+^: 310.05, calc. 310.10.

*Procedure for the synthesis of 4-(4-(3-hydroxyphenyl)thiophen-2-yl)-2-methylphenol* **3**: A solution of 0.15 g (0.48 mmol) of 3b in 8 ml of dry dichloromethane was cooled to -78°C and 2.9 ml of BBr_3_ solution (1M in dichloromethane) was carefully added. The mixture was allowed to warm to room temperature and was stirred over night at 25°C. The reaction was stopped by addition of 10 ml of water and extracted with dichloromethane. The combined organic layer was washed with water and brine, dried over magnesium sulfate and the solvent was removed in vacuo. The crude product was purified by flash column chromatography eluting with ethyl acetate/hexane 1:3 to give 35 mg (0.12 mmol), 25 % of **3** as a white solid. ^1^H NMR (500 MHz, Chloroform-*d*) δ ppm 2.17 (s, 3 H) 6.72 (ddd, *J*=7.80, 2.44, 1.10 Hz, 1 H) 6.82 (d, *J*=8.20 Hz, 1 H) 7.09 - 7.13 (m, 1 H) 7.14 - 7.24 (m, 2 H) 7.33 - 7.38 (m, 1 H) 7.46 (d, *J*=2.21 Hz, 1 H) 7.61 (d, *J*=1.58 Hz, 1 H) 7.66 (d, *J*=1.26 Hz, 1 H) 9.48 (s, 1 H) 9.54 - 9.60 (m, 1 H); ^13^C NMR (126 MHz, Chloroform-*d*) δ ppm 21.19, 118.03, 119.42, 120.22, 122.10, 123.71, 125.80, 129.23, 129.75, 129.95, 133.05, 134.98, 141.77, 147.51, 149.85, 160.70, 162.95; Purity(FID): 98 %, MS(EI), m/z [M]^+^: 282.16, calc. 282.07.

*Procedure for the synthesis of 4-(4-bromothiophen-2-yl)isoquinoline* **47a**: 143 mg (0.83 mmol) of (isoquinolin-4-yl)boronic acid was diluted in a mixture of degassed dioxane/water (5:1) under nitrogen atmosphere. To this solution 200 mg (0.83 mmol) of 2,4-dibromothiophene, 220 mg (2.1 mmol) of Na_2_CO_3_ and 27 mg (0.033 mmol) of tetrakis(triphenylphosphine)palladium(0) were added successively. The mixture was heated to reflux and the reaction progress was monitored by TLC (ALUGRAM SIL G/UV_254_ (Macherey-Nagel)). The reaction was stopped when all starting material was consumed and the crude reaction mixture was poured into water and extracted with ethyl acetate (4x). The combined organic extracts were washed with water and brine, dried over Magnesium sulfate and the solvent was removed under reduced pressure. The crude product was purified by flash column chromatography eluting with ethyl acetate/hexane 1:10 to give 110 mg 46 % of 4-(4-bromothiophen-2-yl)isoquinoline as a solid. ^1^H NMR (500 MHz, Methanol–*d*_4_) δ (ppm) 7.18 – 7.19 (m, 1 H) 7.55 – 7.55 (m, 1 H) 7.66 (ddd, *J*=8.80, 6.90, 1.00 Hz, 1 H) 7.75 (ddd, *J*=8.43, 6.86, 1.42 Hz, 1 H) 8.05 – 8.10 (m, 2 H) 8.38 (s, 1 H) 9.16 (d, *J*=0.63 Hz, 1 H), ^13^C NMR (126 MHz, Methanol–*d*_4_) δ (ppm) 111.35, 124.99, 125.78, 126.95, 129.30, 129.57, 129.80, 131.89, 133.18, 135.22, 140.04, 143.29, 153.92; Purity(FID): 99 %, MS(EI), m/z [M]^+^: 288.90, calc. 288.95.

Analytical data of the ISYNTH Chemspeed compounds synthesized according to the general procedure used for the synthesis of the focused library.

*4-methyl-3-[5-(pyridin-3-yl)thiophen-3-yl]pyridine* **6**: mp 73-74°C; IR (neat) 3050, 1416, 1126, 1022, 798, 698, 616 cm^-1^; ^1^H-NMR (300 MHz, CDCl_3_): *δ* ppm 8.90 (d, *J* = 2.42 Hz, 1H), 8.53 - 8.56 (m, 2H), 8.44 (d, *J* = 5.03 Hz, 1H), 7.89 (td, *J* = 2.00, 7.92 Hz, 1H), 7.39 (d, *J* = 1.49 Hz, 1H), 7.33 (ddd, *J* = 0.75, 4.66, 8.01 Hz, 1H), 7.29 (d, *J* = 1.49 Hz, 1H), 7.19 (d, *J* = 5.03 Hz, 1H), 2.39 (s, 3H). ^13^C-NMR (75 MHz, CDCl_3_): *δ* ppm 20.10, 123.68, 124.18, 125.34, 125.51, 130.02, 132.30, 133.00, 139.33, 140.80, 144.76, 146.88, 148.42, 148.76, 149.57. Purity (UV): 99 %, t_R_: 2.98 min MS (ESI+): *m/z* (%) = 255 (7), 254 (23), 253 (100) [M+H^+^], calc. 253.07.

*2-methyl-5-[5-(pyridin-3-yl)thiophen-3-yl]pyridine* **7**: mp 121-122°C; IR (neat) 3056, 1419, 1125, 1023, 798, 699, 600 cm^-1^; ^1^H-NMR (300 MHz, CDCl_3_): *δ* ppm 2.58 (s, 3H), 7.19 (d, *J* = 8.0 Hz, 1H), 7.32 (dd, *J* = 8.0, 4.8 Hz, 1H), 7.47 (d, *J* = 1.3 Hz, 1H), 7.54–7.61 (m, 1H), 7.77 (dd, *J* = 8.0, 2.4 Hz, 1H), 7.89 (dt, *J* = 8.0, 2.0 Hz, 1H), 8.54 (dd, *J* = 4.8, 1.5 Hz, 1H), 8.76 (d, *J* = 2.2 Hz, 1H), 8.90 (d, *J* = 2.4 Hz, 1H), ^13^C-NMR (75 MHz, CDCl_3_): *δ* ppm 23.93, 121.26, 122.87, 123.31, 123.68, 128.40, 130.02, 133.02, 133.96, 139.90, 141.62, 146.52, 146.75, 148.69, 157.23. Purity (UV): 99 %, t_R_ = 3.03 min; MS (ESI+): m/z (%) = 254 (18), 253 (100) [M+H^+^], 148 (21), 127 (21), calc. 253.07.

*2-methoxy-5-[5-(pyridin-3-yl)thiophen-3-yl]pyridine* **9**: mp 132-133°C; IR (neat) 3071, 1420, 1124, 1030, 1016, 795, 697, 609 cm^-1^; ^1^H-NMR (300 MHz, CDCl_3_): *δ* ppm 3.97 (s, 3H), 6.80 (d, *J* = 8.6 Hz, 1H), 7.32 (dd, *J* = 8.0, 4.8 Hz, 1H), 7.37–7.40 (m, 1H), 7.51–7.57 (m, 1H), 7.79 (dd, *J* = 8.7, 2.5 Hz, 1H), 7.85–7.92 (m, 1H), 8.43 (d, *J* = 2.4 Hz, 1H), 8.54 (dd, *J* = 4.8, 1.3 Hz, 1H), 8.91 (d, *J* = 2.2 Hz, 1H), ^13^C-NMR (75 MHz, CDCl_3_): *δ* ppm 53.55, 110.93, 120.18, 122.88, 123.65, 124.86, 130.10, 132.93, 136.69, 140.02, 141.52, 144.35, 146.91, 148.79, 163.52; Purity: 98 %, *t*_R_: 5.91 min; MS (ESI+): *m/z* (%) = 271 (13), 270 (38), 269 (100) [M+H^+^], calc. 269.07.

*3-ethoxy-5-[5-(pyridin-3-yl)thiophen-3-yl]pyridine* **10**: mp 116-117°C; IR (neat) 3075, 2980, 2937, 2889, 1606, 1505, 1467, 1431, 1329, 1285, 1135, 1042; ^1^H-NMR (500 MHz, CDCl_3_): *δ* ppm 1.39 (t, *J* = 7.1 Hz, 3H,), 4.34 (q, *J* = 7.3 Hz, 2H), 6.82 (d, *J* = 8.5 Hz, 1H), 7.48 (dd, *J* = 7.9, 4.7 Hz, 1H), 7.67 (d, *J* = 1.3 Hz, 1H), 7.85 (d, *J* = 1.3 Hz, 1H), 8.00 (dd, *J* = 8.5, 2.5 Hz, 1H), 8.12 (d, *J* = 7.9 Hz, 1H), 8.42–8.50 (m, 2H), 8.88 (s, 1H), ^13^C-NMR (126 MHz, CDCl_3_): *δ* ppm 15.01, 63.18, 111.94, 121.91, 124.61, 125.66, 126.35, 132.33, 134.96, 138.39, 141.29, 142.05, 145.30, 146.97, 149.03, 164.66; Purity: 96 %, *t*_R_ : 6.68 min, MS (ESI+): *m/z* (%) = 285 (10), 284 (29), 283 (100) [M+H^+^], 169 (7), calc. 283.08.

*3-[4-(pyridin-4-yl)thiophen-2-yl]pyridine* **11**: mp 117-118°C; IR (neat) 3663, 1414, 1126, 1025, 799, 700, 614 cm^-1^; ^1^H-NMR (300 MHz, CDCl_3_): *δ* ppm 8.87 - 8.95 (m, 1H), 8.63 (dd, *J* = 1.49, 5.40 Hz, 2H), 8.56 (dd, *J* = 1.49, 4.84 Hz, 1H), 7.88 - 7.95 (m, 1H), 7.67 (dd, *J* = 1.30, 9.13 Hz, 2H), 7.54 (dd, *J* = 1.86, 3.73 Hz, 2H), 7.37 (ddd, *J* = 0.75, 4.89, 7.96 Hz, 1H). ^13^C-NMR (75 MHz, CDCl_3_): *δ* ppm 120.88 (2C), 122.64, 123.90, 129.93, 133.40, 140.17, 141.90, 142.96, 146.33, 148.47, 149.42 (2C). Purity (UV): 97 %, t_R_ = 2.81 min; MS (ESI+): m/z (%) = 241 (13), 240 (38), 239 (100) [M+H^+^], calc. 239.06.

*2-methyl-4-[5-(pyridin-3-yl)thiophen-3-yl]pyridine* **12**: mp 162-163°C; IR (neat) 3055, 1446, 1124, 1030, 802, 697, 612 cm^-1^; ^1^H-NMR (300 MHz, CDCl_3_): *δ* ppm 8.92 (dd, *J* = 0.93, 2.42 Hz, 1H), 8.53 - 8.59 (m, 2H), 7.90 - 7.96 (m, 1H), 7.62 - 7.70 (m, 2H), 7.41 (d, *J* = 0.56 Hz, 1H), 7.37 - 7.40 (m, 1H), 7.35 - 7.37 (m, 1H), 2.63 (s, 3H); ^13^C-NMR (75 MHz, CDCl_3_): δ ppm 23.57, 118.31, 120.68, 122.75, 123.86, 123.90, 130.00, 133.42, 140.37, 141.83, 143.50, 146.39, 148.50, 148.54, 158.38. *Purity: 99 %,* *t*_R_ = 2.93 min; MS (ESI+): *m/z* (%) = 255 (6), 254 (18), 253 (100) [M+H^+^], calc. 253.07.

*5-[5-(pyridin-3-yl)thiophen-3-yl]pyridine-3-carbonitrile* **13**: mp 217-218°C; IR (neat) 3093, 2228, 1456, 1136, 1022, 808, 705, 617; ^1^H NMR (300 MHz, CDCl_3_): *δ* ppm 9.08 (d, *J* = 2.24 Hz, 1H), 8.93 (d, *J* = 2.24 Hz, 1H), 8.83 (d, *J* = 2.05 Hz, 1H), 8.60 (dd, *J* = 1.40, 4.75 Hz, 1H), 8.13 - 8.18 (m, 1H), 7.88 - 7.95 (m, 1H), 7.62 - 7.63 (m, 1H), 7.61 (d, *J* = 0.93 Hz, 1H), 7.37 (dd, *J* = 4.84, 7.82 Hz, 1H); ^13^C NMR (75 MHz, CDCl_3_): *δ* ppm 91.4, 110.28, 116.38, 122.36, 123.38, 123.78, 129.51, 131.50, 133.11, 136.23, 137.45, 143.01, 147.01, 149.37, 150.65; Purity: 95 %, t_R_ : 5.15 min; MS (ESI+): *m/z* (%) = 305 (12) [M+ACN+H^+^], 266 (6), 265 (20), 264 (100) [M+H^+^], calc. 264.05.

*3-[5-(pyridin-3-yl)thiophen-3-yl]-5-(trifluoromethyl)pyridine* **14**: mp 171-172°C; IR (neat) 3056, 1468, 1119, 1023, 802, 697, 608; ^1^H NMR (300 MHz, CDCl_3_): *δ* ppm ^1^H NMR (300 MHz, CHLOROFORM-d) d 9.00 (s, 1H), 8.85 (s, 1H), 8.76 (s, 1H), 8.51 (d, *J* = 4.47 Hz, 1H), 8.03 (s, 1H), 7.85 (d, *J* = 7.82 Hz, 1H), 7.55 (d, *J* = 2.61 Hz, 2H), 7.28 (dd, *J* = 4.84, 7.82 Hz, 1H); ^13^C NMR (75 MHz, CDCl_3_): *δ* ppm 122.57, 122.99, 123.38 (d, *J* = 272.7 Hz, 1C), 123.72, 126.86 (q, *J* = 33.1 Hz, 1C), 129.62, 130.28 (q, *J* = 3.4 Hz, 1C), 131.22, 133.05, 138.21, 142.62, 145.07 (q, *J* = 3.9 Hz, 1C), 146.94, 149.18, 150.54; Purity: 98 %, t_R_: 6.75 min; MS (ESI+): *m/z* (%) = 309 (26), 308 (70), 307 (100) [M+H^+^], calc. 307.04.

*3-chloro-5-[5-(pyridin-3-yl)thiophen-3-yl]pyridine* **16**: mp 158-159°C; IR (neat) 3102, 1457, 1112, 1064, 1015, 800, 702, 626 cm^-1^; ^1^H NMR (300 MHz, Methanol-d_4_): *δ* ppm 8.92 (d, *J* = 1.68 Hz, 1H), 8.76 (d, *J* = 1.86 Hz, 1H), 8.57 (dd, *J* = 1.49, 4.66 Hz, 1H), 8.52 (d, *J* = 2.05 Hz, 1H), 7.85 - 7.94 (m, 2H), 7.59 (d, *J* = 1.49 Hz, 1H), 7.56 (d, *J* = 1.49 Hz, 1H), 7.34 (ddd, *J* = 0.75, 5.22, 8.01 Hz, 1H); ^13^C NMR (75 MHz, Methanol-d_4_): *δ* ppm 122.66, 123.71, 129.71, 132.24, 132.29, 133.01, 133.09, 138.37, 142.34, 145.25, 146.95, 147.30, 149.12. 1C not det.; Purity: 95, t_R_ : 6.01 min; MS (ESI+): *m/z* (%) = 276 (21), 275 (93) [M+H^+^], 274 (52), 273 (100) [M+H^+^], calc. 273.02.

*methyl 5-[5-(pyridin-3-yl)thiophen-3-yl]pyridine-3-carboxylate* **18**: mp 210-211°C; ^1^H-NMR (300 MHz, CDCl_3_): δ ppm 4.00 (s, 3H), 7.36 (ddd, *J* = 8.0, 4.8, 0.8 Hz, 1H), 7.62 (d, *J* = 1.5 Hz, 1H), 7.66 (d, *J* = 1.5 Hz, 1H), 7.93 (ddd, *J* = 7.9, 2.4, 1.6 Hz, 1H), 8.50 (t, *J* = 2.1 Hz, 1H), 8.58 (dd, *J* = 4.8, 1.5 Hz, 1H), 8.93 (dd, *J* = 2.4, 0.8 Hz, 1H), 9.05 (d, *J* = 2.4 Hz, 1H), 9.16 (d, *J* = 2.1 Hz, 1H), ^13^C-NMR (75 MHz, CDCl_3_): δ ppm 52.58, 122.59, 122.75, 123.74, 126.14, 129.81, 131.04, 133.06, 134.31, 138.78, 142.38, 147.02, 149.15, 149.42, 150.98, 165.68; Purity: >99%; *t*_R_ : 4.99 min, MS (ESI+): *m/z* (%) = 338 (5) [M+ACN+H^+^], 299 (16), 298 (46), 297 (100) [M+H^+^], calc. 297.06.

*Procedure for the synthesis of compound 4-(5-(pyridin-3-yl)thiophen-3-yl)isoquinoline* **21**: To a solution of 73 mg (0.42 mmol) (isoquinolin-4-yl)boronic acid in 10 ml of Dioxane and 4 ml of water was successive added 2.5 eq. of Na_2_CO_3_ (1.3 mmol, 137 mg), 4 mol % of Tetrakis(triphenylphosphine)Palladium(0) (0.017 mmol, 19.4 mg), 100 mg (0.42 mmol) of 3-(4-bromothiophen-2-yl)pyridine under nitrogen atmosphere. The mixture was stirred and heated to reflux over-night. The reaction progress was monitored by TLC analysis on ALUGRAM SIL G/UV_254_ (Macherey-Nagel). The crude mixture was cooled to room temperature, washed with water and brine and extracted with ethyl acetate. The combined organic layers were dried over Magnesium sulfate and concentrated in vacuo. The crude product was purified by flash column chromatography eluting with ethyl acetate/hexane 1:5 to yield 68 mg (64 %) of **21** as a light yellow solid. mp 126-127°C; IR 3044, 1578, 1342, 1124, 1021, 799, 701, 612 cm^-1^; ^1^H NMR (500 MHz, Methanol-*d*_4_) δ ppm 9.19 (d, *J* = 0.95 Hz, 1H), 8.88 (dd, *J* = 0.79, 2.36 Hz, 1H), 8.47 (dd, *J* = 1.58, 5.04 Hz, 1H), 8.45 (s, 1H), 8.11 - 8.15 (m, 2H), 8.07 - 8.10 (m, 1H), 7.78 - 7.82 (m, 1H), 7.72 (d, *J* = 1.26 Hz, 1H), 7.69 - 7.71 (m, 1H), 7.67 (d, *J* = 1.26 Hz, 1H), 7.47 (ddd, *J* = 0.95, 4.89, 8.04 Hz, 1H); ^13^C NMR (126 MHz, Methanol-*d*_4_) δ ppm 125.52, 125.64, 126.89, 127.88, 129.08, 129.46, 130.01, 132.07, 132.82, 135.06, 135.56, 139.60, 141.96, 142.57, 147.11, 149.20, 153.01, 1 C not detected; Purity (FID): 95.6 %, t_R_: 9.75 min; MS (EI), *m/z* [M]^+^: 288.00 calc.: 288.072.

*5-[5-(pyridin-3-yl)thiophen-3-yl]pyrimidine* **22**: mp 130-131°C; IR (neat) 3059, 1442, 1131, 1020 ,809, 702, 617 cm^-1^; ^1^H NMR (300 MHz, Methanol-d_4_): *δ* ppm 7.35 (dd, *J* = 8.0, 4.8 Hz, 1H), 7.56–7.65 (m, 2H), 7.86–7.95 (m, 1H), 8.58 (dd, *J* = 4.8, 1.3 Hz, 1H), 8.89–8.95 (m, 1H), 8.95–9.01 (m, 2H), 9.16 (s, 1H), ^13^C NMR (75 MHz, Methanol-d_4_): *δ* ppm 122.22, 122.83, 123.72, 129.20, 129.57, 133.06, 136.18, 142.81, 146.97, 149.22, 154.08, 157.43. 1C not det; Purity: 99 %, t_R_ : 3.84 min; MS (ESI+): *m/z* (%) = 242 (9), 241 (25), 240 (100) [M+H^+^], calc. 240.05.

*2-chloro-5-[5-(pyridin-3-yl)thiophen-3-yl]pyridin-3-amine* **23**: mp 210-212°C; IR (neat) 3300, 3184, 1418, 1084, 1044, 1026, 800, 703, 590 cm^-1^; ^1^H-NMR (300 MHz, Methanol-d_4_): δ ppm 8.90 (dd, *J* = 0.79, 2.36 Hz, 1H), 8.49 (dd, *J* = 1.58, 5.04 Hz, 1H), 8.15 (ddd, *J* = 1.58, 2.52, 8.20 Hz, 1H), 8.01 (d, *J* = 2.21 Hz, 1H), 7.88 (d, *J* = 1.26 Hz, 1H), 7.79 (d, *J* = 1.58 Hz, 1H), 7.49 - 7.53 (m, 2H); ^13^C-NMR (75 MHz, Methanol-d_4_): *δ* ppm 120.93, 123.79, 124.79, 125.70, 132.20, 133.15, 134.10, 135.06, 135.48, 140.65, 142.34, 142.94, 147.05, 149.21; Purity: 99 %; *t*_R_: 4.76 min; MS (ESI+): *m/z* (%) = 291 (5), 290 (35) [M+H^+^], 289 (14), 288 (100) [M+H^+^], 229 (14), calc. 288.03.

*3-(4-{3-methyl-3H-imidazo[4,5-b]pyridin-6-yl}thiophen-2-yl)pyridine* **24**: mp 212-213°C; IR (neat) 3060, 1402, 1122, 1021, 799, 702, 619 cm^-1^; ^1^H-NMR (300 MHz, CDCl_3_): *δ* ppm 3.96 (s, 3H, CH_3_), 7.33–7.47 (m, 1H), 7.53 (d, *J* = 1.5 Hz, 1H, Ar_m_-H), 7.67 (d, *J* = 1.5 Hz, 1H, Ar_m_-H), 7.89–8.05 (m, 1H), 8.15 (s, 1H), 8.28 (d, *J* = 2.1 Hz, 1H), 8.57 (dd, *J* = 5.0, 1.6 Hz, 1H), 8.72 (d, *J* = 2.1 Hz, 1H), 8.94 (dd, *J* = 2.4, 0.8 Hz, 1H), ^13^C-NMR (75 MHz, CDCl_3_): *δ* ppm 30.03, 121.48, 123.69, 124.01, 125.24, 127.08, 130.47, 133.66, 134.74, 140.64, 141.31, 143.19, 145.33, 146.12, 146.60, 147.97; Purity: 99 %; *t*_R_ : 3.68 min; MS (ESI+): *m/z* (%) = 294 (6), 293 (21) [M+H^+^], 168 (89), 147 (100), calc. 293.08.

*3-[5-(pyridin-3-yl)thiophen-3-yl]pyridin-1-ium-1-olate* **25**: mp 168-169°C; IR (neat) 3069, 1432, 1277, 1160, 1012, 783, 708, 614 cm^-1^; ^1^H NMR (300 MHz, CDCl_3_): *δ* ppm 8.87 (d, *J* = 1.49 Hz, 1H), 8.51 - 8.60 (m, 2H), 8.12 - 8.20 (m, 1H), 7.87 (ddd, *J* = 1.49, 2.33, 7.92 Hz, 1H), 7.55 - 7.58 (m, 1H), 7.53 - 7.55 (m, 1H), 7.51 (dd, *J* = 1.21, 8.10 Hz, 1H), 7.29 - 7.38 (m, 2H); ^13^C NMR (75 MHz, CDCl_3_): *δ* 163.7, 149.0, 146.7, 142.5, 137.5, 136.9, 136.9, 134.6, 133.1, 129.5, 126.0, 124.1, 123.7, 123.4, 122.3; Purity: 98 , t_R_ : 3.21 min; MS (ESI+): *m/z* (%) = 256 (17), 255 (100) [M+H^+^], calc. 255.05.

*1-methyl-4-[5-(pyridin-3-yl)thiophen-3-yl]-1,2-dihydropyridin-2-one* **26**: mp 178-179°C; IR (neat) 3084, 1475, 1129, 1023, 804, 704, 605 cm^-1^; ^1^H-NMR (300 MHz, CDCl_3_): *δ* ppm 8.87 (d, *J* = 2.24 Hz, 1H), 8.54 (dd, *J* = 1.30, 4.84 Hz, 1H), 7.86 - 7.93 (m, *J* = 1.90, 1.90 Hz, 1H), 7.60 (d, *J* = 1.30 Hz, 1H), 7.56 (d, *J* = 0.93 Hz, 1H), 7.30 - 7.40 (m, 2H), 6.85 (d, *J* = 1.86 Hz, 1H), 6.46 (dd, *J* = 1.96, 6.98 Hz, 1H), 3.56 (s, 3H); ^13^C-NMR (75 MHz, CDCl_3_): δ ppm 37.39, 105.01, 115.57, 122.63, 123.89, 124.26, 129.92, 133.39, 138.32, 139.69, 141.63, 145.65, 146.32, 148.44, 163.43; Purity: 99, t_R_ = 3.69 min; MS (ESI+): *m/z* (%) = 271 (9), 270 (29), 269 (100) [M+H^+^], calc. 269.07.

*3-[4-(2,3-dihydro-1-benzofuran-5-yl)thiophen-2-yl]pyridine* **27**: mp 136-137°C; IR (neat) 2965, 1436, 1123, 1021, 804, 704, 614 cm^-1^; ^1^H-NMR (300 MHz, CDCl_3_): *δ* ppm 3.26 (t, *J* = 8.7 Hz, 2H), 4.61 (t, *J* = 8.7 Hz, 2H), 6.83 (d, *J* = 8.2 Hz, 1H), 7.27–7.39 (m, 3H), 7.45 (s, 1H), 7.56 (d, *J* = 1.1 Hz, 1H), 7.85–7.97 (m, 1H), 8.53 (dd, *J* = 4.7, 1.3 Hz, 1H), 8.91 (d, *J* = 1.9 Hz, 1H); ^13^C-NMR (75 MHz, CDCl_3_): δ ppm 29.67, 71.43, 109.49, 119.22, 123.04, 123.50, 123.67, 126.33, 127.70, 128.32, 130.48, 132.93, 140.67, 143.61, 146.72, 148.40, 159.75; Purity: 99 %, t_R_ = 6.72 min; MS (ESI+): *m/z* (%) = 282 (9), 281 (24), 280 (100) [M+H^+^], calc. 280.07.

*3-[4-(1,3-thiazol-4-yl)thiophen-2-yl]pyridine* **28**: mp 123-124°C; IR (neat) 3076, 1441, 1121, 1023, 800, 700, 613 cm^-1^; ^1^H-NMR (300 MHz, CDCl_3_): *δ* ppm 8.89 - 8.95 (m, 1H), 8.82 - 8.86 (m, 1H), 8.53 (dd, *J* = 1.49, 4.84 Hz, 1H), 7.89 (ddd, *J* = 1.49, 2.61, 8.01 Hz, 1H), 7.72 - 7.80 (m, 2H), 7.40 - 7.46 (m, 1H), 7.31 (ddd, *J* = 0.93, 4.84, 8.01 Hz, 1H); ^13^C-NMR (75 MHz, CDCl_3_): δ ppm 112.43, 122.51, 123.01, 123.62, 130.08, 132.90, 137.23, 141.10, 146.81, 148.66, 151.72, 152.92; Purity: 99 %; *t*_R_ = 4.69 min; MS (ESI+): *m/z* (%) = 247 (14), 246 (29), 245 (100) [M+H^+^], calc. 245.01.

*3-[4-(1-methyl-1H-imidazol-5-yl)thiophen-2-yl]pyridine* **31**: mp 97-98°C; IR (neat) 3083, 1568, 1414, 1129, 1021, 808, 700, 611 cm^-1^; ^1^H NMR (300 MHz, CDCl_3_): *δ* 8.89 (s, 1H), 8.56 (d, *J* = 4.28 Hz, 1H), 7.89 (d, *J* = 7.64 Hz, 1H), 7.52 (s, 1H), 7.40 (s, 1H), 7.27 - 7.38 (m, 2H), 7.18 - 7.24 (m, 1H), 3.75 (s, 3H); ^13^C NMR (75 MHz, CDCl_3_): *δ* ppm 149.0, 147.0, 141.2, 133.0, 131.3, 129.8, 128.5, 124.5, 123.7, 122.3, 32.8.; Purity: 99 %, t_R_ : 2.66 min; MS (ESI+): *m/z* (%) = 243 (15), 243 (100) [M+H^+^], calc. 242.07.

*3-(4-{3H,8aH-imidazo[1,2-a]pyridin-3-yl}thiophen-2-yl)pyridine* **32**: mp 146-147°C; IR (neat) 2981, 1419, 1154, 1022, 807, 708, 608 cm^-1^; ^1^H-NMR (500 MHz, Methanol-d_4_): *δ* 8.89 (d, *J* = 1.58 Hz, 1H), 8.60 (td, *J* = 1.10, 6.94 Hz, 1H), 8.48 (dd, *J* = 1.42, 4.89 Hz, 1H), 8.13 (ddd, *J* = 1.58, 2.36, 8.04 Hz, 1H), 7.83 (d, *J* = 1.26 Hz, 1H), 7.82 - 7.83 (m, 1H), 7.81 (d, *J* = 1.58 Hz, 1H), 7.63 (td, *J* = 0.99, 9.06 Hz, 1H), 7.47 (ddd, *J* = 0.79, 4.81, 7.96 Hz, 1H), 7.41 (ddd, *J* = 1.26, 6.62, 9.14 Hz, 1H), 7.06 (dt, *J* = 1.26, 6.78 Hz, 1H); ^13^C NMR (126 MHz, Methanol-d_4_): *δ* ppm 15.05, 117.63, 123.05, 124.45, 125.67, 125.84, 126.05, 127.51, 131.25, 131.75, 131.85, 135.13, 142.41, 146.67, 147.13, 149.35; Purity: 99 %, *t*_R_ = 2.93 min; MS (ESI+): *m/z* (%) = 280 (14), 279 (33), 278 (100) [M+H^+^], calc. 280.08.

*3-chloro-5-[5-(1,3-oxazol-5-yl)thiophen-3-yl]pyridine* **34**: mp 118-119°C; IR (neat) 3083, 1469, 1108, 1024, 817, 738, 624 cm^-1^; ^1^H-NMR (300 MHz, CDCl_3_): *δ* ppm 8.59 - 8.66 (m, 1H), 7.89 (s, 1H), 7.79 - 7.86 (m, 1H), 7.51 - 7.56 (m, 1H), 7.47 - 7.51 (m, 1H), 7.37 (d, *J* = 8.38 Hz, 1H), 7.29 (s, 1H); ^13^C-NMR (75 MHz, CDCl_3_): *δ* ppm 121.88, 121.91, 122.84, 124.35, 129.82, 131.31, 136.20, 138.13, 146.29, 147.17, 150.19, 150.30; Purity: 98 %, t_R_ = 7.10 min; MS (ESI+): *m/z* (%) = 266 (8), 265 (63) [M+H^+^], 264 (23), 263 (100) [M+H^+^], calc. 263.0.

*3-methyl-5-[5-(1,3-oxazol-5-yl)thiophen-3-yl]pyridine* **35**: mp 117-118°C; IR (neat) 3073, 1421, 1140, 1032, 810, 706, 632 cm^-1^; ^1^H-NMR (300 MHz, CDCl_3_): *δ* ppm 2.37 (s, 3H), 7.26 (s, 1H), 7.46 (s, 1H), 7.55 (s, 1H), 7.65 (s, 1H), 7.86 (s, 1H), 8.38 (s, 1H), 8.66 (s, 1H), ^13^C-NMR (75 MHz, CDCl_3_): *δ* ppm 18.37, 121.37, 121.66, 123.17, 130.29, 130.79, 133.17, 133.99, 139.62, 144.72, 146.51, 149.18, 150.05; Purity: 95 %, t_R_ = 3.91 min; MS (ESI+): *m/z* (%) = 245 (7), 244 (18), 243 (100) [M+H^+^], calc. 243.05.

*5-[5-(1,3-oxazol-5-yl)thiophen-3-yl]pyrimidine* **36**: mp 177-178°C; IR (neat) 3112, 1447, 1098, 1007, 839, 717, 633 cm^-1^; ^1^H-NMR (300 MHz, CDCl_3_): *δ* ppm 7.31 (s, 1H), 7.55–7.61 (m, 2H), 7.91 (s, 1H), 8.96 (s, 2H), 9.16 (s, 1H); ^13^C-NMR (75 MHz, CDCl_3_): *δ* ppm 122.11, 122.38, 122.53, 128.89, 131.82, 135.80, 146.09, 150.29, 154.09 (2C), 157.51; Purity: 99 %, t_R_ = 4.85 min; MS (ESI+): *m/z* (%) = 232 (10), 231 (27), 230 (100) [M+H^+^], calc. 230.03.

*2-(methylsulfanyl)-5-[5-(1,3-oxazol-5-yl)thiophen-3-yl]pyrimidine* **37**: mp 166-167°C; IR (neat) 3075, 1414, 1103, 998, 815, 627 cm^-1^; ^1^H-NMR (300 MHz, CDCl_3_): *δ* ppm 2.60 (s, 3H), 7.30 (s, 1H), 7.48 (d, *J* = 1.2 Hz, 1H), 7.52 (d, *J* = 1.2 Hz, 1H), 7.90 (s, 1H), 8.72–8.79 (m, 2H); ^13^C-NMR (75 MHz, CDCl_3_): *δ* ppm 14.19, 121.38, 122.03, 122.33, 124.02, 131.58, 135.97, 146.23, 150.25, 154.40 (2C), 171.61; Purity: 93 %, t_R_ = 6.90 min; MS (ESI+): *m/z* (%) = 278 (14), 277 (23), 276 (100) [M+H^+^], calc. 276.02.

*5-[4-(1H-pyrazol-4-yl)thiophen-2-yl]-1,3-oxazole* **38**: mp 175-176°C; IR (neat) 3095; 1426; 1098; 997; 810; 723; 601 cm^-1^; ^1^H NMR (500 MHz, DMSO-d_6_) δ 12.94 (br. s., 1H), 8.43 (s, 1H), 7.92 - 8.19 (m, 2H), 7.75 (d, *J* = 1.58 Hz, 1H), 7.65 (d, *J* = 1.58 Hz, 1H), 7.50 (s, 1H); ^13^C NMR (126 MHz, DMSO-d_6_) δ ppm 151.3, 146.1, 134.7, 129.0, 123.9, 121.3, 118.4, 116.3; Purity: 95 %, t_R_ = 4.95 min; MS (ESI+): *m/z* (%) = 220 (8), 219 (14), 218 (100) [M+H^+^], calc. 218.03.

*4-[5-(1,3-oxazol-5-yl)thiophen-3-yl]isoquinoline* **39**: mp 179-180°C; IR (neat) 3072, 1393, 1103, 1021, 800, 688, 637 cm^-1^; ^1^H-NMR (300 MHz, CDCl_3_): *δ* ppm 9.26 (d, *J* = 0.75 Hz, 1H), 8.56 (s, 1H), 8.05 - 8.08 (m, 1H), 8.01 - 8.04 (m, 1H), 7.90 (s, 1H), 7.70 - 7.77 (m, 1H), 7.61 - 7.69 (m, 1H), 7.54 (d, *J* = 1.49 Hz, 1H), 7.46 (d, *J* = 1.30 Hz, 1H), 7.30 (s, 1H); ^13^C-NMR (75 MHz, CDCl_3_): *δ* ppm 121.74, 124.36, 124.57, 126.45, 127.40, 127.49, 128.04, 128.40, 130.25, 130.88, 134.11, 138.26, 142.79, 146.62, 150.11, 152.45; Purity: 99 %, t_R_ = 4.86 min; MS (ESI+): *m/z* (%) = 281 (10), 280 (34), 279 (100) [M+H^+^], calc. 279.05.

*5-[5-(1,3-oxazol-5-yl)thiophen-3-yl]-1H-indole* **40**: mp 161-162°C; IR (neat) 3142, 1429, 1114, 1039, 812, 666, 595 cm^-1^; ^1^H-NMR (300 MHz, CDCl_3_): *δ* ppm 8.30 (br. s., 1H), 7.89 (s, 2H), 7.66 (d, *J* = 1.49 Hz, 1H), 7.45 - 7.49 (m, 1H), 7.41 - 7.45 (m, 1H), 7.39 (d, *J* = 1.30 Hz, 1H), 7.27 (s, 1H), 7.23 - 7.26 (m, 1H), 6.61 (dt, *J* = 1.02, 2.10 Hz, 1H); ^13^C-NMR (75 MHz, CDCl_3_): *δ* ppm 103.00, 111.36, 118.53, 119.16, 121.08, 121.16, 124.30, 125.03, 127.38, 128.31, 129.67, 135.37, 144.38, 147.18, 149.85; Purity: 97 %, *R*_f_ = 0.68 / *t*_R_ = 7.22 min; MS (ESI+): *m/z* (%) = 269 (11), 268 (33), 267 (100) [M^+^], calc. 267.05.

*5-[4-(5-chloropyridin-3-yl)thiophen-2-yl]pyrimidine* **42**: mp 210-211°C; IR (neat) 3066, 1427, 1107, 1024, 838, 715, 630 cm^-1^; ^1^H-NMR (300 MHz, CDCl_3_): *δ* ppm 7.40 (d, *J* = 8.4 Hz, 1H), 7.62 (d, *J* = 5.0 Hz, 2H), 7.86 (dd, *J* = 8.1, 2.3 Hz, 1H), 8.66 (s, 1H), 9.00 (s, 2H), 9.17 (s, 1H), ^13^C-NMR (75 MHz, CDCl_3_): *δ* = 123.30, 123.73, 124.45, 128.13, 129.83, 136.28, 138.17, 138.99, 147.24, 150.51, 153.52 (2C), 157.80; Purity: 90 %, t_R_ = 6.44 min; MS (ESI+): *m/z* (%) = 277 (8), 276 (44) [M+H^+^], 275 (19), 274 (100) [M+H^+^], calc. 274.01.

*5-[4-(5-methylpyridin-3-yl)thiophen-2-yl]pyrimidine* **43**: mp 165-166°C; IR (neat) 3041, 1423, 1115, 1030, 789, 714, 630 cm^-1^; ^1^H-NMR (300 MHz, CDCl_3_): *δ* ppm 2.40 (s, 3H), 7.59 (d, *J* = 1.5 Hz, 1H), 7.66 (d, *J* = 1.5 Hz, 1H), 7.68–7.74 (m, 1H), 8.38–8.45 (m, 1H), 8.69 (d, *J* = 1.9 Hz, 1H), 8.97–9.04 (m, 2H), 9.15 (s, 1H); ^13^C-NMR (75 MHz, CDCl_3_): *δ* ppm 18.41, 122.79, 124.01, 128.31, 130.29, 133.27, 134.07, 137.60, 140.48, 144.77, 149.37, 153.44, 157.61; Purity: 96 %, t_R_ = 3.54 min; MS (ESI+): *m/z* (%) = 256 (5), 255 (19), 254 (100) [M+H^+^], calc. 254.07.

*5-[4-(pyrimidin-5-yl)thiophen-2-yl]pyrimidine* **44**: mp 135-136°C; IR (neat) 3108, 1412, 1187, 1036, 839, 717, 631 cm^-1^; ^1^H-NMR (500 MHz, CDCl_3_): *δ* ppm 7.67 (d, *J*=1.3 Hz, 1 H) 7.70 (d, *J*=1.3 Hz, 1 H) 9.00 (s, 2 H) 9.01 (s, 2 H) 9.19 (s, 1 H) 9.20 (s, 1 H); ^13^C-NMR (126 MHz, CDCl_3_): *δ* ppm 123.31, 123.97, 127.96, 128.93, 136.65, 138.71, 153.59 (2C), 154.17 (2C), 157.70, 157.94; Purity: 97 %, t_R_ : 3.39 min; MS (ESI+): *m/z* (%) = 243 (5), 242 (16), 241 (100) [M+H^+^], calc. 241.05.

*2-(methylsulfanyl)-5-[5-(pyrimidin-5-yl)thiophen-3-yl]pyrimidine* **45**: mp 221-222°C; IR (neat) 3083, 1409, 1171, 1033, 851, 720, 634 cm^-1^; ^1^H-NMR (300 MHz, CDCl_3_): *δ* ppm 9.28 (s, 1H), 9.17 (s, 1H), 9.03 (s, 2H), 8.58 (s, 1H), 8.02 - 8.10 (m, 2H), 7.71 - 7.78 (m, 1H), 7.64 - 7.71 (m, 1H), 7.63 (d, *J* = 1.30 Hz, 1H), 7.58 (d, *J* = 1.49 Hz, 1H); ^13^C-NMR (75 MHz, CDCl_3_): *δ* ppm 14.21, 122.80, 123.20, 123.99, 128.05, 136.77, 138.41, 153.54 (2C), 154.43 (2C), 157.83, 171.79; Purity: 95 %, t_R_ : 6.26 min; MS (ESI+): *m/z* (%) = 289 (12), 288 (15), 287 (100) [M+H^+^], calc. 287.03.

*4-[5-(pyrimidin-5-yl)thiophen-3-yl]isoquinoline* **46**: mp 235-236°C; IR (neat) 3083, 1468, 1108, 1025, 816, 715, 624 cm^-1^; ^1^H-NMR (300 MHz, CDCl_3_): *δ* ppm 7.58 (d, *J* = 1.3 Hz, 1H), 7.63 (d, *J* = 1.3 Hz, 1H), 7.65–7.78 (m, 2H), 8.02–8.10 (m, 2H), 8.58 (s, 1H), 9.03 (s, 2H), 9.17 (s, 1H), 9.28 (s, 1H), ^13^C-NMR (75 MHz, CDCl_3_): *δ* ppm 124.29, 125.96, 127.22, 127.50, 128.14, 128.40, 128.44, 131.01, 134.13, 136.62, 137.03, 139.07, 142.75, 152.54, 153.54 (2C), 157.63; Purity: 99 %, t_R_ = 4.37 min; MS (ESI+): *m/z* (%) = 292 (7), 291 (19), 290 (100) [M+H^+^], calc. 290.07.

*Procedure for the synthesis of compound* **47**: 47 mg (0.38 mmol) 3-Pyridylboronic acid was dissolved in degassed dioxane/H_2_O (5:1) under nitrogen atmosphere. 110 mg (0.38 mmol) of 4-(4-bromothiophen-2-yl)isoquinoline, 100 mg (0.95 mmol) of Na_2_CO_3_ and 22 mg (0.02 mmol) of Tetrakis(triphenylphosphine)Palladium(0) were added and the mixture was stirred under reflux. The reaction progress was monitored by TLC analysis on ALUGRAM SIL G/UV_254_ (Macherey-Nagel) until the whole starting material was consumed. After completion of the reaction, the crude product was washed with water and brine, extracted with ethyl acetate and purified by flash column chromatography eluting with ethyl acetate/hexane 1:3 to give 44 mg (40.3 %) of **47** as a light yellow solid; mp 99-100°C; IR(neat) 3088, 3043, 1567, 1322, 1121, 1022, 799, 702, 616 cm^-1^; ^1^H NMR (500 MHz, Methanol-*d*_4_) δ ppm 9.21 (s, 1H), 8.91 (d, *J* = 2.21 Hz, 1H), 8.50 (s, 1H), 8.46 (dd, *J* = 1.10, 4.89 Hz, 1H), 8.24 (d, *J* = 8.51 Hz, 1H), 8.15 (d, *J* = 7.88 Hz, 2H), 7.97 (d, *J* = 0.95 Hz, 1H), 7.80 - 7.84 (m, 1H), 7.73 (d, *J* = 7.25 Hz, 1H), 7.71 (d, *J* = 0.95 Hz, 1H), 7.47 (dd, *J* = 5.04, 7.88 Hz, 1H); ^13^C NMR (126 MHz, Methanol-*d*_4_) δ ppm 124.72, 125.33, 125.56, 127.86, 128.22, 129.27, 129.56, 129.92, 133.12, 133.26, 135.44, 135.68, 140.19, 140.21, 143.29, 147.84, 148.69, 153.63; Purity (FID): 99 %, t_R_: 9.97 min; MS (EI), m/z [M]^+^: 288.08, calc. 288.07.

**2) Construction of the pET45b-Dyrk1A-cd expression plasmid.**

Firstly, cDNA was produced from human placenta total RNA using the “Transcriptor High Fidelity cDNA Synthesis kit” (Roche Applied Science) and oligo-dT18 primer according to the instructions of the manufacturer. Then a nested primer strategy was employed to amplify the cDNA sequence coding for the catalytic domain of Dyrk1A by PCR using the “Expand High Fidelity PCR^PLUS^ System” (Roche Applied Science. A 0.5 µl aliquot of the cDNA synthesis mixture was first amplified in a final volume of 25 µl using 0.4 µM each primer (D1A-for, TGACTTGATCAAAACATACAAGCAT; D1A-rev: ATGAGTTTCAACAGTGACCTGTGTA), 0.2 mM of each dNTP, and 1 x Expand High Fidelity buffer2. The following cycling conditions were used on an Eppendorf Mastercycler Gradient (Eppendorf AG, Germany): initial denaturation: 94 °C, 2 min; then 10 cycles of denaturation at 94 °C, 15s; annealing at 63 °C, 30s; elongation at 72 °C, 1.5 min. The cycling was continued for 15 cycles of denaturation at 94 °C, 15s; annealing at 63°C, 30s; elongation at 72 °C, 1.5 min plus 5 sec increment for each new cycle, followed by a final elongation at 72 °C for 7 min. The PCR products were purified from residual primers and primer dimers using the GeneJET™ PCR Purification Kit (Fermentas). Half of the purified product was applied to a second round of PCR using the nested primer pair D1Afor-thr-age (GAACCGGTCTGGTGCCGCGCGGATCCAGTCATAAGAAGGAACGGAAGG) and D1Arev-hind (CGTAAGCTTCTATTCATCAGCTGTTTTCTTGAAGAAAC), carrying the AgeI and HindIII restriction sites, respectively, and in the D1Afor-thr-age primer a thrombin cleavage site was also included. Now the following cycling conditions were used:

initial denaturation at 94 °C, 2 min; then 10 cycles of denaturation at 94 °C, 15s; annealing at 55 °C, 30s; elongation at 72 °C, 1 min 10 sec. The cycling was continued for 5 cycles of denaturation at 94 °C, 15s; annealing at 57°C, 30s; elongation at 72 °C, 1 min 10 sec plus 5 sec increment for each new cycle, followed by a final elongation at 72 °C for 7 min.

The final PCR product was purified by agarose gel electrophoresis, cutting out of the gel band containing the 1.1 kb product, and finally isolation of the DNA using the GeneJET™ Gel Extraction Kit (Fermentas). The PCR product was cloned into the pCR2.1 vector which is part of the TOPO® TA cloning Kit (Life Technologies), transformed into E.coli TOP10 cells, and single colonies were picked from LB agar plates containing 100 µg/ml ampicillin after growing overnight. The single colonies were grown in 5 ml LB/amp cultures overnight at 37 °C with shaking, and the plasmid DNA isolated the next day using the Plasmid Mini Kit from Qiagen. The plasmids were sequenced, and the cDNA with the correct sequence was cut out from the pCR2.1 vector using the AgeI and HindIII restriction enzymes (New England Biolabs) and subcloned using T4 DNA ligase (Fermentas) into the pET45b(+) vector (Novagen) which had been digested with the same restriction enzymes.

1. **Literature**

1. Thompson AL, Kabalka GW, Akula MR, Huffman JW (2005) The Conversion of Phenols to the Corresponding Aryl Halides Under Mild Conditions. Synthesis (Stuttg) 2005: 547–550. Available: http://www.thieme-connect.de/DOI/DOI?10.1055/s-2005-861791. Accessed 4 March 2013.
